# Incidence of hospital-acquired pressure ulcers in patients with "minimal risk" according to the "Norton-MI" scale

**DOI:** 10.1371/journal.pone.0227052

**Published:** 2020-01-08

**Authors:** Isabel Díaz-Caro, Soledad García Gómez-Heras

**Affiliations:** 1 International Doctoral School, Rey Juan Carlos University, Madrid, Spain; 2 Nurse of Hospital University Severo Ochoa, Madrid, Spain; 3 Basic Health Science Department, Health Science Faculty, Rey Juan Carlos University, Madrid, Spain; Northwestern University Feinberg School of Medicine Galter Health Sciences Library, UNITED STATES

## Abstract

**Introduction:**

Pressure ulcers (PUs) nowadays are a major health problem in society, associated with increased morbidity and increased health care costs. The incidence of HAPU is an indicator of health care quality.

**Objective:**

To describe the profile of patients with minimal risk on the Norton-MI scale who developed PUs during hospitalization, and to identify the incidence of hospital-acquired pressure ulcers (HAPU).

**Methods:**

Retrospective cohort study conducted between 2014 and 2017.

**Study population:**

Patients over 18 years of age classified as "minimum risk" according to Norton-MI, admitted to acute hospital units of the Severo Ochoa University Hospital—Madrid-Spain. Patients were classified as patients with hospital-acquired pressure ulcers (PWHAPU) if they developed one or more new PU during their hospitalization.

**Variables:**

Sociodemographic variables, hospitalization units, Morton-MI score and characteristics of the risk factors of HAPU were studied.

**Results:**

The risk of PU was evaluated in 5530 patients, being 1260 patients classified as "minimum risk", with a median of 16 points in the Norton-MI scale. The average age was 76 years old and 52.5% were women. Principal causes of admission: traumatological pathologies (20.8%) and cardiovascular pathologies (20%). 129 HAPU were diagnosed in 112 patients, implying an incidence of HAPU of 8.89% (CI95%: 7.44–10.59). 106 PWHAPU (94.6%) presented up to 6 risk factors. The excess pressure and altered skin sensibility were identified as statistically significant risk factors as predictive factors of HAPU. In terms of severity, 55% of the HAPU were category I and 42.6% were category II, mainly with anatomical sacro-coxygeal location. In 65.2% of the patients the HAPU appeared in the first week of hospitalization.

**Conclusion:**

In our study the incidence of HAPU in patients classified as minimum risk with Norton-MI scale was 8.89%. This elevated risk suggests for future investigations to develop new validity studies of the Norton-MI scale and to maintain a continuo training of professionals in the knowledge of PU risk assessment scales for their safe application in the patients, since it directs the practice of care in the prevention of PUs. It would be advisable to specially control the risk of PUs in care units, mainly in the first week of their hospital stay to minimize the HAPU incidence.

## Introduction

Pressure ulcers (PUs) are considered chronic cutaneous wounds as they involve a prolonged and slow healing time, with a strategy of cure by second intention and sometimes recurrent in its appearance. They are usually located in the skin and underlying tissues, over a bony prominence, caused by sustained pressure over time, deformation, friction and/or the combination of these.(European Pressure Ulcer Advisory Panel—EPUAP) [1–4]. These injuries are evidence of a major public health problem, as well as for the health system due to its epidemiological, economic and socio-family impact [5].

Pam Hibbs in 1987 described this health situation as "An epidemic under the sheets", and stated that 95% of PUs are preventable, but studies such as the one carried out in the United Kingdom (UK) in 5 acute hospitals of the NHS in 2012, concluded that only 43% of HAPU were preventable, that is why "these results suggest that the figure that 95% of the PUs are preventable is questionable, at least with regard to hospital-acquired pressure damage". [6,7].

Currently, 65% of PUs are of nosocomial origin, primarily affecting people over 65 years of age, with limitations especially in the functional patterns: nutritional-metabolic, elimination, activity-exercise and cognitive-perceptual, and presenting risk factors that contribute to the development of PUs as advanced age, excess pressure and impaired skin sensitivity [8–12]. Therefore, the reduction and prevention of cases are one of the main concerns in health organizations.

Risk assessment is a key aspect for the prevention of PUs. The identification of patients with risk from the first contact with the hospital environment, by validated PURAS [13–16], as Braden, Gosnell, Norton, and Waterlow [17], is the first step to follow to guide nurses in therapeutic decision making. As recommended by the clinical practice guidelines (CPG) and the panel of experts (EPUAP) [18–23]. However, there is no consensus among experts and practitioners on the best way to perform PUs risk assessment and which is the ideal PURAS, existing studies with evidence regarding this aspect.[24,25]. The National Group for the Study and Advice of the PU (NGSAPU) "Grupo Nacional para el estudio y asesoramiento en ulceras por Presión y Heridas Crónicas (GNEAUPP)*"* in Spain recommends focusing the effort on lines of research that validate scales already built.

The quality indicators established as prevention and care for PUs are useful for assessing care for PUs in care units and health organizations. The incidence rate of HAPUs can provide a useful indicator in the hospital setting to assess the success of prevention protocols. Epidemiological studies carried out by The National Pressure Ulcer Advisory Panel (NPUAP) publishes for America an incidence of 7% although it varies depending on the type of patient [26,27]. In Europe it is established around 18.1% with differences between countries [28], in Spain, in the latest national studies carried out by the GNEAUPP 2013 and 2018 of Prevalence of PUs, it was striking that around 7% of patients with PUs were classified as "low risk" with some PURAS– 2013, [29] and in 2018 the prevalence of PUs in hospitals of medium complexity was 6.6% [30].

Having detected in clinical practice a considerable frequency of patients rated as having "minimal risk" with the appearance of HAPU, we believe it is necessary to dimension the problem, by knowing the incidence of HAPU, as well as the characteristics of these patients, to question the tool used to rate the risk and the cut-off point of minimum risk assessment. All of this offers great potential for future studies on HAPU in hospitalized adult patients in acute care units. This will allow evidence-based protocols to be developed, in order to reduce the incidence of HAPU.

**Fig 1:**
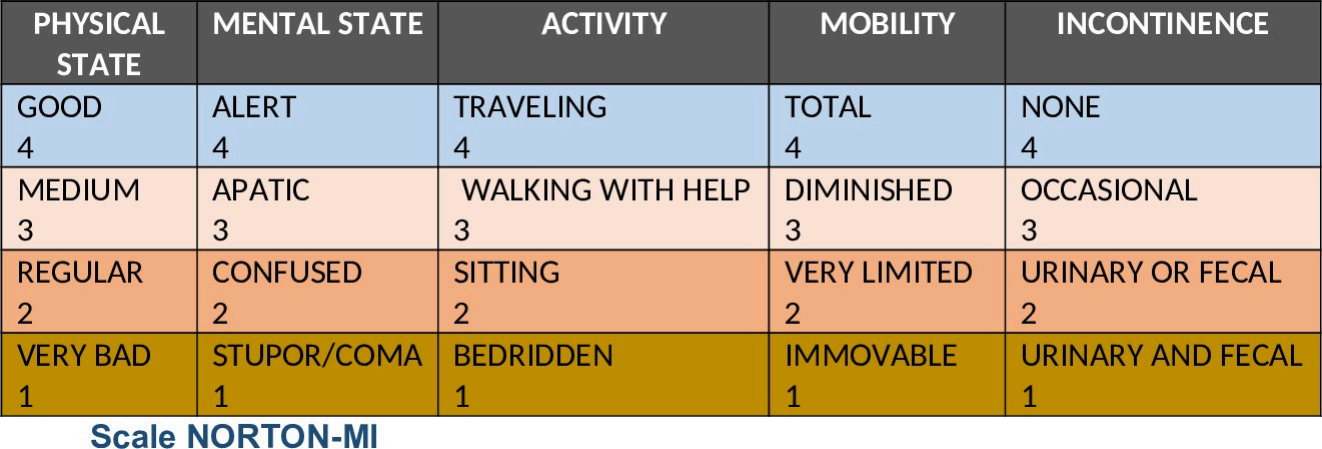
NORTON Modified by INSALUD (Norton-MI) is a modified version of the scale developed by Norton et al. (1962) and was adapted by INSALUD in 1996 (National Institute for Health, 1996 [15].

The objective of this study was to describe the profile of adult patients hospitalized in acute care units with assessment on the PURAS Norton scale Modified by the National Health System (Norton-MI) (Fig 1), as "minimal risk" that have developed HAPU, and to identify the incidence of HAPU in these patients.

## Methods

### Study setting

Retrospective cohort study. Study period from January 1, 2014 to December 31, 2017. It was carried out at the Severo Ochoa University Hospital in Madrid, Spain, of medium complexity—Level II of care, belonging to the public network of hospitals of the Madrid Health Service (SERMAS), with capacity of 412 beds, 291 belonging to the 6 hospitalization units of nursing care monitored for this study: 3 internal medicine, 2 vascular surgery and general digestive surgery and 1 polyvalent of geriatrics and traumatology.

### Data collection

In the hospital, nurses carry out their care activity guided by the following hospital protocols: 1.- "Protocol for the Reception of Patients at the Hospital" and 2.- "Protocol for the Prevention, Communication and Monitoring of PUs". Compliance applies to all nurses/staff belonging to hospitalization units, responsible for the care of the patient.

The data have been collected retrospectively. Primary Sources: Clinical History (CH): comprising the demographic data and periods of hospital stay of the patient. Nurse clinical forms: (1) "Nursing Assessment on Admission", which includes the items for the recording of socio-demographic and clinical admission data (age, sex, hospitalization units, primary medical diagnosis) and the risk assessment of the PUs Norton-MI scale, performed by the Staff nurse, on the first day of admission to the care unit (initial valoration). The nurse documents the evaluation point of the Norton-MI on the clinical form, and activates the best practice of attention alerts for skin care. (2) "PU prevention and follow-up communication", includes the items for recording data related to the PUs on prevention, communication and follow-up, carried out by the patient's Staff nurse each time he or she identifies the appearance of a HAPU and cares for the patient during the hospital stay.

### Defining the study population

**Fig 2:**
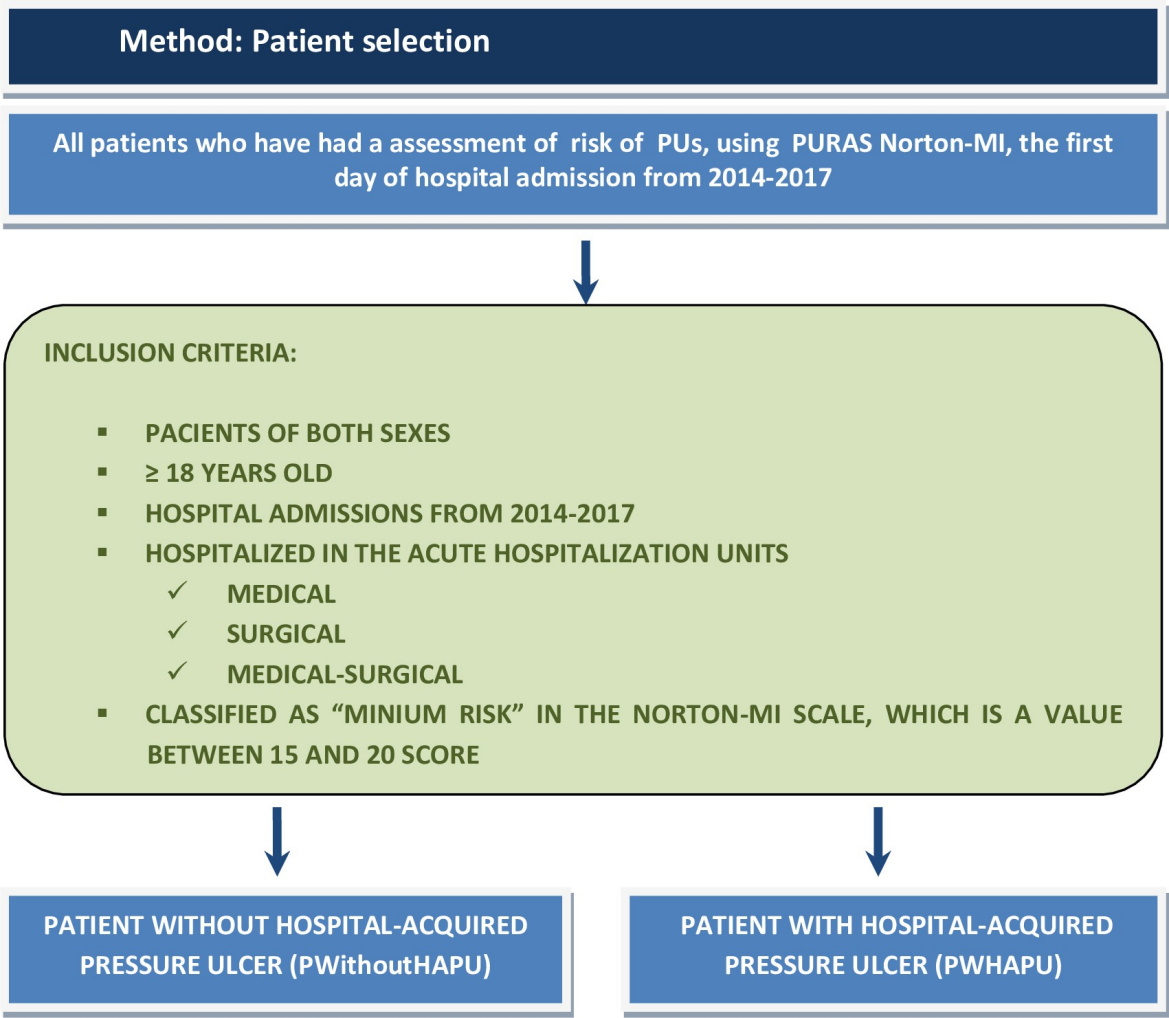
Method: Inclusion criteria for the selection of patients.

The study population is all adult patients of both sexes over 18 years of age, hospitalized in nursing care units, internal medicine physicians, vascular surgery and general digestive surgery, and polyvalent geriatrics and traumatology, which were classified as "minimum risk" in the PURAS Norton-MI, by the nurse staff on the 1st day in the hospitalization unit (Fig 2). Patients were classified as patients with HAPU (PWHAPU) if they developed one or more new category I ulcers or more pressure ulcers during their hospitalization. The studies of incidence based on care units cannot avoid differences in their composition with respect to the existing specialties in the defined populations, which is why we have calculated the incidence of HAPU in the three areas of monitored care units, hoping that it will help to compare the incidence as a reference value with other care areas and countries.

### Definition of hospital-acquired pressure ulcers incidence

The main outcome of interest is the estimation of the incidence of HAPU with its 95% confidence interval (CI95%). Incidence has been defined as "the proportion of people in a given population who initially did not have PU and who developed them in a given period of time", as well as "those patients who, having previously had PU, develop new lesions" and it was calculated using the following formula: (number of patients who developed at least one HAPU during the study period/total study population) × 100) [31–33].

### Statistical analysis

The statistical analysis carried out using Excel and Statistical Package for the Social Sciences (SPSS) IBM version 22 for Windows.

The qualitative variables are described by means of absolute frequencies and percentages, and the quantitative variables are described by means of the mean and the standard deviation or median and interquartile range.

An bivariate analysis is performed to study the possible factors associated with the development of HAPU calculated from Pearson's Ӽ^2^ test and estimating relative risk (RR) with 95% confidence interval as a measure of association. A p-value of p<0.05 was considered statistically significant.

### Ethics statement

This study has been favourably certified by the Research Commission and by the Clinical Research Ethics Committee (CREC) of the Severo Ochoa University Hospital in Madrid.

The CREC, both in its composition and in the standard work processes, complies with the standards (CPMP/ICH/135/95) and with Order SAS 3470/2009 of 16 December. For this study the CREC and the Research Committee considered that the necessary requirements for the suitability of the protocol in relation to the objectives of the study were met and that the risks and subject too. As a retrospective study, the CREC considered it unnecessary to obtain informed consent from patients. All the data has been obtained from the CH and the nursing clinical forms, and had been exported to an Excel file by anonymized download. All the information obtained as a result of this study will be considered confidential in accordance with the provisions of Law 15/1999 of 13 December on the Protection of personal data, and Law 3/2018 of 5 December on the Protection of Personal Data and guarantee of digital rights. [34–36].

## Results

**Fig 3:**
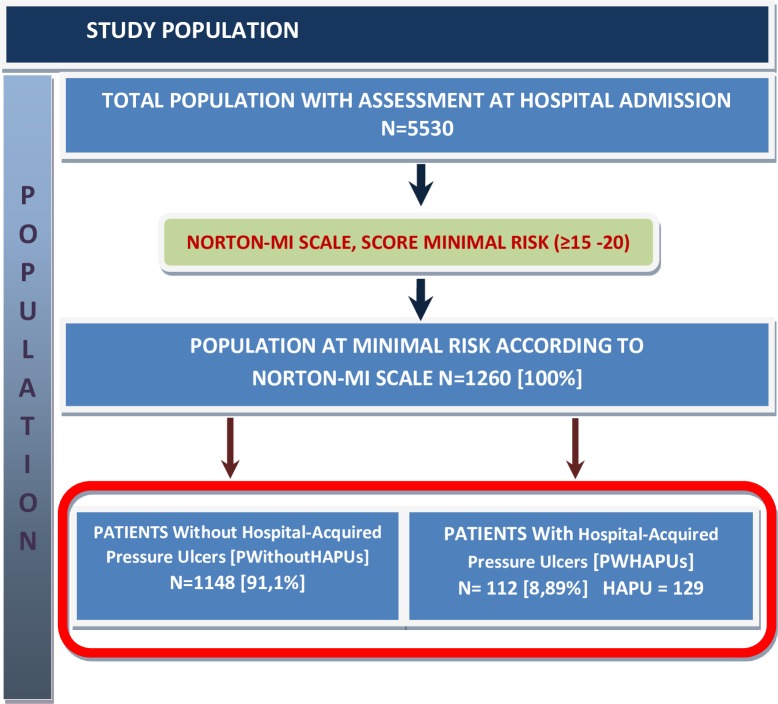
Description of the population under study.

In the study period, 5530 patients were evaluated for PU risk and 1260 were classified as "minimal risk" according to the PURAS Norton-MI (Fig 3).

### Characteristics of "minimal risk" patients and incidence of HAPU

**Fig 4:**
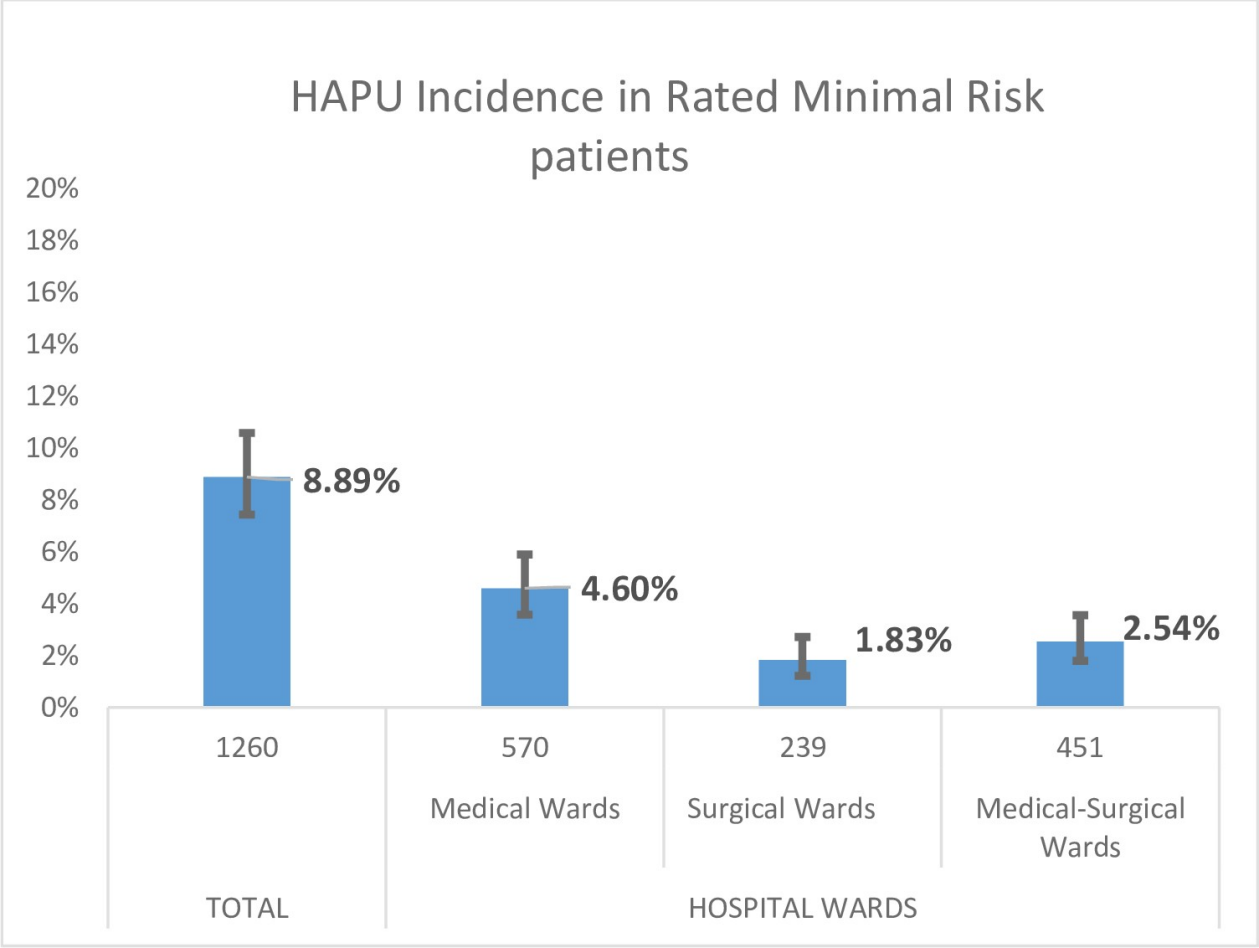
Incidence of HAPU in minimal risk rated patients per nursing care unit. Bars represent 95% confidence interval.

The Table 1 shows the characteristics of the population classified as "minimal risk" n = 1260 patients. The mean age was 76 years (SD 14.3; Max 102; Min 19); 598 (47.5%) were men and 662 (52.5%) women. HAPU was diagnosed in 112 patients, implying an incidence of HAPU in patients of "minimal risk" of 8.89% (CI95%: 7.44% - 10.59%). Fig 4 shows the incidence of HAPU per nursing care unit, being this incidence greater in medical units.

**Table 1:** Description of the characteristics of the population classified as "minimal risk", patient with HAPU (PWHAPU) and patient without HAPU (PWithoutHAPU).

		Minimal Risk Level Norton-MI Scale (15–20)					
		Total Population		PWithoutHAPU		PWHAPU	
		n = 1260		n = 1148		n = 112	
		n	%	n	%	n	%
**Gender**							
	**Female**	662	(52.5)	610	(53.1)	52	(46.4)
**Age (years)**							
	**mean (SD)**	76	(14.3)	76	(14.5)	76,8	(11.7)
**Age (years) grouped**							
	**< 65**	234	(18.6)	215	(18.7)	19	(17.0)
	**66–79**	671	(53.3)	613	(53.4)	58	(51.8)
	**> 80**	355	(28.2)	320	(27.9)	35	(31.3)
**Hospitalization Wards**							
	**Medical Wards**	570	(45.2)	512	(44.6)	58	(51.8)
	**Surgical Wards**	239	(19)	216	(18.8)	23	(20.5)
	**Medical-Surgical Wards**	451	(35.8)	420	(36.6)	31	(27.7)
**Principal Diagnostic / Reason for admission**							
	**Cardiovascular**	252	(20.0)	237	(20.6)	15	(13.4)
	**Respiratory**	205	(16.3)	176	(15.3)	29	(25.9)
	**Urinary**	85	(6.7)	77	(6.7)	8	(7.1)
	**Nervous System**	99	(7.9)	93	(8.1)	6	(5.4)
	**Endocrine/Diabetes**	20	(1.6)	16	(1.4)	4	(3.6)
	**Tumoral**	69	(5.5)	62	(5.4)	7	(6.3)
	**Sepsis/infection**	81	(6.4)	72	(6.3)	9	(8)
	**Digestive**	180	(14.3)	165	(14.4)	15	(13.4)
	**Traumatology**	262	(20.8)	244	(21.3)	18	(16.1)
	**Others**	7	(0.6)	6	(0.6)	1	(0.9)
**Norton-MI Scale**							
	**Median (IQR = Q1,Q3)**	16	(15.18)	17	(16.18)	16	(15.17)
**Nº Risk Factors for pacients (grouped)**							
	**0–5 RF**	827	(65.6)	752	(65.5)	75	(67.0)
	**6–10 RF**	433	(34.4)	396	(34.5)	37	(33.0)
**Identified Risk Factors**							
	**Alt. mobility**	899	(71.3)	827	(72)	72	(64.3)
	**Excess Pressure**	429	(34.0)	359	(31.3)	70	(62.5)
	**Alt. Level of consciousness**	105	(8.3)	95	(8.3)	10	(8.9)
	**Alt. activity**	852	(67.6)	770	(67.1)	82	(73.2)
	**Alt nutrition**	728	(57.8)	695	(60.5)	33	(29.5)
	**Alt body temperature**	257	(20.4)	229	(19.9)	28	(25.0)
	**Alt skin sensitivity**	471	(37.4)	417	(36.3)	54	(48.2)
	**Secondary effects of treatment**	554	(44)	534	(46.5)	20	(17.9)
	**Incontinence (faecal-urinary)**	470	(37.3)	440	(38.3)	30	(26.8)
	**Age ≥ 65 years**	1041	(82.6)	944	(82.2)	97	(86.6)
**Days of hospital stay**							
	**Median (IQR = Q1,Q3)**	9	(6,14)	9	(5,13)	15	(9.29)
**Days of hospital stay (grouped)**							
	**< 7**	417	(33.1)	405	(35.3)	12	(10.7)
	**7–14**	538	(42.7)	497	(43.3)	41	(36.3)
	**15–21**	182	(14.4)	160	(13.9)	22	(19.6)
	**22–28**	60	(4.8)	51	(4.4)	9	(8.0)
	**> 28**	63	(5.0)	35	(3.0)	28	(25.0)

Patient with hospital-acquired pressure ulcer (PWHAPU); Patient without hospital-acquired pressure ulcer (PWithoutHAPU)

### Characteristics of the PWHAPU patients and HAPU description

Analyzing the PWHAPU sample with "minimum risk" (n = 112), the mean age was 76.8 (SD 11.75) (Max 98, Min 49) and 52 cases were women (46.4%). The most frequent primary medical diagnoses were respiratory pathologies (25.9%), followed by traumatological pathologies (16.1%). The PWHAPU were mainly admitted to medical units (51.8%). Hospital stay length reported a median of 15 days (IQR 9, 29), and the median onset of HAPU was 6 days (IQR 3, 14). 106 PWHAPU (94,6%) provided up to 6 risk factors (RFs), advanced age, altered activity and mobility, excess pressure, altered skin sensitivity and altered nutrition.

A total of 129 HAPU are diagnosed in 112 patients (Table 2) 86.6% of patients developed a single HAPU, being 71 HAPU (55%) of category I, 55 (42.6%) of category II, and 3 HAPU (2.3%) of category III, mainly the most affected anatomical Location areas were sacro-coxygeal in 61 HAPU (47.3%) and heels in 39 lesions (30.2%), in the order shown (Table 3). The first HAPU (HAPU-1) has Category I, II and III injuries; HAPU-2 has Category I and II injuries; and HAPU-3 has Category I injuries. In all of them, The sacrum-coccyx zone is the anatomical location that most frequently presents these ulcers (Table 4).

**Table 2:** Describes the frequency of the number of HAPU per patient and HAPU declaration time in PWHAPU.

		PWHAPU	
Nº of HAPU developed in the patient		n = 112	%
	**1**	97	(86.6)
	**2**	13	(11.6)
	**3**	2	(0.2)
**First day of the declaration of HAPU**			
	**Median (IQR = Q1,Q3)**	6	(3,14)
**First day of the declaration of HAPU (grouped)**			
	**≤ 7**	63	(56.3)
	**8–15**	24	(21.4)
	**16–21**	5	(4.5)
	**> 21**	20	(17.9)

Patient with hospital-acquired pressure ulcer (PWHAPU)

**Table 3:** General description of HAPU, frequency by categories and anatomical location.

		HAPU—1		HAPU—2		HAPU—3		TOTAL	
		n	%	n	%	n	%	n	%
**CATEGORIES/STAGE**		**112**	** **	**15**	** **	**2**		**129**	
	**CATEGORY I**	58	(51.8)	11	(73.3)	2	(100.0)	71	(55.0)
	**CATEGORI II**	51	(45.5)	4	(26.7)			55	(42.6)
	**CATEGORY III**	3	(2.7)					3	(2.3)
**ANATOMIC LOCATION**		** **							
	**Sacrum-coccyx**	57	(50.9)	3	(20.0)	1	(50.0)	61	(47.3)
	**Heel**	31	(27.7)	7	(46.7)	1	(50.0)	39	(30.2)
	**Maleolos**	9	(8.1)	3	(20.0)			12	(9.4)
	**Trocanter**	2	(1.8)					2	(1.6)
	**Areas of the head**	1	(0.9)					1	(0.8)
	**Areas of lower limbs**	2	(1.8)					2	(1.6)
	**Areas of superior limbs**	4	(3.6)	1	(6.7)			5	(3.9)
	**Other**	6	(5.4)	1	(6.7)			7	(5.5)

First HAPU(HAPU-1); Second HAPU (HAPU-2); Third HAPU (HAPU -3)

**Table 4:** Detailed description of HAPU by category and anatomical location.

		HAPU—1		HAPU—2		HAPU—3	
CATEGORY I		n = 112	%	n = 15	%	n = 2	%
**Anatomical location**	**Sacrum-coccyx**	27	(38.0)	1	(1.4)	1	(1.4)
	**Heel**	19	(26.8)	6	(8.5)	1	(1.4)
	**Maleolos**	4	(5.6)	2	(1.8)		
	**Trocanter**	1	(1.4)				
	**Areas of the head**	1	(1.4)				
	**Areas of superior limbs**	2	(1.8)	1	(1.4)		
	**Other**	4	(52.5)	1	(1.4)		
**CATEGORY II**							
**Anatomical location**	**Sacrum-coccyx**	28	(50.9)	2	(3.6)		
	**Heel**	11	(20.0)	1	(1.8)		
	**Maleolos**	5	(9.1)	1	(1.8)		
	**Trocanter**	1	(1.8)				
	**Areas of lower limbs**	2	(3.6)				
	**Areas of superior limbs**	2	(3.6)				
	**Other**	2	(3.6)				
**CATEGORY III**							
**Anatomical location**	**Sacrum-coccyx**	2	(66.7)				
	**Heel**	1	(33.3)				

First HAPU(HAPU-1); Second HAPU (HAPU-2); Third HAPU (HAPU-3)

Regarding the study of risk factors in Table 5 it is shown a bivariate analysis. Risk factors associated with the presence of HAPU in patients with “minimal risk” assessment on the Norton-MI scale. We found a statistically significant association between the development of a HAPU and RFs, excess pressure (RR = 3.23, CI95% = 2.24–4.65); altered skin sensivity (RR = 1.56 CI95% 1.1–2.22) for a value of p<0.000. Regarding the hospitalization time of patients with HAPU, a statistically significant association was obtained with hospital stays >7 days (RR = 4.12 CI 95% 2.29–7.41 p<0.001); >14 days (RR = 3.29. CI 59% 2.31–4.68 p<0.001) and >21 days (RR = 4.38 CI95% 3.09–6.19 p<0.001). No significant association was found in hospitalization units.

**Table 5:** Shows a bivariate analysis. Risk factors associated with the presence of HAPU in patients with “minimal risk” assessment on the Norton-MI scale.

	BIVARIATE ANALYSIS			
	CARACTERISTIC	RR	CI 95%	p < value
**Gender**		** **	** **	** **
	**Male**	1,28	0,90–1,82	0,175
**Risk Factors (grouped)**		** **	** **	** **
	**6–10 RF**	0,97	0,63–1,42	0,904
**Risk Factors**				
	**Alt mobility (yes)**	0,72	0,50–1,04	0,083
	**Alt activity (yes)**	1,31	0,88–1,96	0,185
	**Alt nutrition (yes)**	0,31	0,21–0,45	< 0,001
	**Excess pressure (yes)**	3,23	2,24–4,65	< 0,001
	**Age ≥ 65 years (yes)**	1,36	0,81–2,30	0,243
	**Alt level of conciousness (yes)**	1,08	0,58–2,00	0,811
	**Alt body temperature (yes)**	1,30	0,87–1,95	0,205
	**Incontinence (faecal-urinary) (yes)**	0,61	0,41–0,92	0,016
	**Secondary effects of treatment (yes)**	0,28	0,17–0,44	< 0,001
	**Alt of skin sensitivity (yes)**	1,56	1,10–2,22	0,013
**Days of hospital stay**				
	**> 7 days**	4,12	2,29–7,41	<0,001
	**> 14 days**	3,29	2,31–4,68	<0,001
	**> 21 days**	4,38	3,09–6,19	<0,001
**Hospitalization Wards**				
	**Medical Wards**	1,30	0,91–1,85	0,145
	**Surgical Wards**	1,1	0,71–1,71	0,658
	**Medical-Surgical Wards**	0,69	0,46–1,02	0,061

Values for relative risk: RR (p<0,05 95% Confidence interval)

## Discussion

HAPUs are injuries considered as one of the indicators of quality in health organizations and therefore their knowledge demonstrates the level of quality in health care. They represent a public health and patient safety problem, and are believed to be avoidable between 55–70% [37]. Whether PUs are avoidable is the main topic of discussion in health institutions, due to the social, health, and legal implications related to the prevention of these injuries, so it is a major challenge for nursing professionals, being the objective to be able to control it and as far as possible eradicate it. The Wound, Ostomy and Continence Nurses Society (WOCN) [17], in its position paper on avoidable PUs injuries versus unavoidable, “claim that it is reasonable to say that not all PUs are avoidable, given the clinical complexity and the large number of RFs observed in patients, all this leads to the development of these lesions. In our sample the patients presented a profile very close to that defined by the WOCN Society, but we cannot determine whether the HAPU in our patients could have been avoidable. The data presented here suggest we question: the cut-off point of the value of “minimum risk” in the Norton-MI scale, and its validity, and the training of nursing professionals in the control and registration of PUs risk prevention care in hospitalized patients.

There are many studies aimed at identifying the incidence and prevalence of these lesions, but few studies have found epidemiological results on the incidence of HAPU in adult patients classified as "minimal risk" with validated PURAS. In Spain, the last study carried out by GNEAUPP in 2013, [29] showed an incidence of 7% in patients with low risk assessment, and our study reported a higher than expected result, an incidence of HAPU of 8.89% produced in the area of acute nursing care (medical, surgical and medico-surgical), in patients classified as "minimal risk" with Norton-MI scale in the hospital setting. This finding highlights the need for caution to report comparable epidemiological data on the incidence of HAPU along with the structure of patients with "minimum risk" assessment with validated scales (PURAS), because, although the sample size is big, in both the study in Spain and in ours is large, there is no reliability of the time of patient assessment, as it is not explicitly referenced in the study conducted in Spain. It is difficult to find explicit reference to the methodology used by health professionals on how, and at what point in time, the level of PU risk in the patient has been assessed. The WOCN Society for the prevention of PUs supports the following measures: to carry out an initial evaluation in the first 24 hours of admission, a continuous evaluation of the risk of PUs, and to apply preventive care based on evidence. In our patients, despite the initial and continuous risk evaluation of PUs, an incidence of 8.89% HAPU was obtained. We believe that this result could be due to two causes: an inadequate interpretation of the risk assessment scale Norton-MI, and the validity of the cut-off point of the lowest risk on the Norton-MI scale (15–20 points).

The research, over time, has directed its efforts to validate or verify the validity and reliability of PURAS such as Braden, Norton among others; some of these studies have revealed that some PURAS have low validity [38,39]. In our hospital Staff nurses carry out the evaluation of the risk of PU of the patients using the PURAS Norton–MI, this is an integrated part of nursing practice for many health care organizations in Spain, and in the Community of Madrid, with a total and subscale score that directs preventive interventions in the development of PUs. Although its validity has been described in adults and elderly people in acute environments, its evidence or recommendation is C3 (low)[15], as it shows difficulty in the evaluation of some subscales as "Physical State" causing discrepancies or ambiguities and, consequently, puts the professional in doubt of carrying out an imprecise or incorrect evaluation. These aspects could explain why in our setting, patients who have developed HAPU obtained a minimal risk assessment [14,15,40,41], and as a consequence of this underestimation of the risk in the first 24 hours since the patient’s hospital admission, has been determinated the therapeutic decision of the professional not to start early the application of preventive interventions, according to the protocol for prevention and monitoring of PUs. On the other hand, the Norton scale was already modified because several studies considered that the original produced false negatives, particularly with a “high risk” of developing PU which were not diagnosed as such, This allows us to question its validity of the cut-off point of the “minimum risk”. Therefore, we suggest new research on the predictive capacity of PURAS Norton-MI in large data sets that could yield evidence for better individualization of preventive interventions.

RFs such as advanced age > 65 years, excess pressure, malnutrition, immobility, inactivity, incontinence and altered skin sensitivity, are RFs that have been the target of many research studies [17,42–44] demonstrating their value as predictors of the risk of HAPU, and therefore their presence is directly noticed in the development of HAPU. Our results show association in the following factors: excess pressure, alteration of skin sensitivity and development of HAPU, but no predictors of HAPU were found in the RFs: alteration in nutrition, side effects of medical treatment (oncological cytostatics toxics corticosteroids), incontinence (fecal and/or urinary). These discrepancies found in contrast to the published literature, make us think that they may be a consequence of the limitations presented by retrospective studies, and the quality of data recorded is not as expected, because, in care units, they undergo continuous changes that are beyond the researchers' control. Another cause may be due to the subjectivity in the measurement of subscales “physical state and incontinence” on the part of the professions because they are not objectively defined, they raise discrepancies and ambiguities that hindered its application in a safe way due to the doubts it generates, this can the professional who applies it in the situation of having to decide randomly in which group the subscale includes the patients [15].

An association was also found in the days of hospital stay and the development of HAPU, with this association increasing exponentially as the days of hospitalization increase. This result is supported by other published studies that state that patients with PUs had longer average stays [45].

With respect to acute care units, numerous works report an association with presenting PUs and being hospitalized in internal medicine and surgical medical care units [8,12,46,47] as they are considered risk units and HAPU prodictors. In our study the majority of patients with HAPU were hospitalized in medical units, and although association was not found with respect to acute medical and surgical care units, we find that this may be a consequence of the type of public hospital and the characteristics of its. units/patients because they combine or change functionality (medical/surgical) according to the care demand, and with respect to the workload of nurses (higher patient/bed rotation). These characteristics are supported by studies such as the one carried out in the US, in 215 hospitals in California, Washington and Oregon, in medical-surgical units where it was concluded that the characteristics of the units/patients were powerful predictors of HAPU although they are generally not modifiable, they are difficult to address in order to reduce HAPU [48].

This suggests that more research is needed to understand the variation of acute care units (medical, surgical, and medico-surgical) between hospitals. For example, the types of HAPU, the specific comorbidities, and the hospital setting (acute medical/critical care unit), may be beneficial. In addition, studying administrative characteristics such as staffing, patient/bed rotation, the staff skills combination, and HAPU prevention competencies could be considered as part of future research.

### Strength and limitation of study

The main strength of the study is the size of the sample obtained during the 4 consecutive years of the study period, we believe this gives it the necessary validity, and will be useful in future comparisons. A limitation of this study the was retrospective data collection, although hospital protocols normalize nursing activity on prevention, communication and follow-up of PUs, there may be undocumented or incomplete or even underestimated information in the HAPU.

## Conclusions

This study, revealed an incidence of 8.89% of HAPU in adult patients valued of “minimum risk” in the medical and surgical acute care units. Being this an incidence higher than expected, and taking into account that patients are valued as having minimal risk, new validity studies of the Norton-MI scale are suggested as lines for researchers and nursing clinicians, and its cut-off point in the minimum risk assessment; more research to understand the variation of acute care hospitalization units among hospitals, the specific comorbidities, and their staff-related administrative characteristics (staffing, staff/bed rotation, care skills and competencies, and PUs follow-up); we propose a special control over the prevention, communication, and follow-up of HAPU in medical and surgical acute care units, mainly in the first week of hospitalization; we also believe it is necessary the continuous training and specialization of professionals in the knowledge of the PURAS for its safe application in the patients, since it leads the assistance practice in the prevention of the PUs.

## Supporting information

S1 File Statistics tables A-D.
(DOCX)

S2 File Logistical regression. (DOCX)

S1 Table Statistics table. (DOCX)
