# Supplementary material for: Incidence of hospital-acquired pressure ulcers in patients with "minimal risk" according to the "Norton-MI" scale
Source: PLoS One. 2020 Jan 8;15(1):e0227052. doi: 10.1371/journal.pone.0227052 (PMC6948734; doi:10.1371/journal.pone.0227052)
Supplement: S1 File — Statistics tables A-D. (DOCX) [file pone.0227052.s001.docx]

## CROSSTABS

/TABLES=n_ulcera BY category

/FORMAT=AVALUE TABLES

/CELLS=COUNT

/COUNT ROUND CELL.

**Cross tables**

Notes

Output created

Comments Entry

01-MAY-2019 15:44:05

Facts

Set of

Filter

C: Users. Diaz\Desktop\TESIS DOCUMENTS\SPSS DATA BASES TESIS\ELIA 2016 2017

2018\data UPP 129 revised elia isa .sav

Data_set1

<none>

Weighting <none>

Segment <none>

N of rows in the file of

129

Management of lost value Definition of

absence

User-defined missing values are treated as lost.

Cases used

The statistics for each table are based on all cases with valid data in the ranges specified for all variables in each table.

Syntax CROSSTABS

/TABLES=n_ulcera BY category

/FORMAT=AVALUE TABLES

/CELLS=COUNT

/COUNT ROUND CELL.

ResourcesTime of 00:00:00,00

Time 00:00:00,01

Dimensions 2

Boxes 131029

Case Processing Summary

|  | Cases | | | | | |
| --- | --- | --- | --- | --- | --- | --- |
|  | Valid | | Lost | | Total | |
|  | N | Percentage | N | Percentage | N | Percentage |
| n_ulcera * CATEGORY_ 1st HAPU | 129 | 100,0% | 0 | 0,0% | 129 | 100,0% |

**n_HAPU_*CATEGORY _ HAPU-1_ cross-tabulation**

Counting

|  |  | CATEGORY _1ª HAPU | | | | |  | |
| --- | --- | --- | --- | --- | --- | --- | --- | --- |
|  |  | CATEGORY 1 |  | CATEGORY 2 |  | CATEGORY 3 |  |  |
| n_HAPU | 1 |  | 58 |  | 51 | 3 |  | 112 |
|  | 2 |  | 11 |  | 4 | 0 |  | 15 |
|  | 3 |  | 2 |  | 0 | 0 |  | 2 |
| Total |  |  | 71 |  | 55 | 3 |  | 129 |

# Custom tables

Notes

Output created Comments

Entrance

01-MAY-2019 15:46:29

Facts

Set of

Filter

C: Users. Diaz\Desktop\TESIS DOCUMENTS\SPSS DATA BASES TESIS\ELIA 2016 2017

2018\data UPP 129 revised elia isa .sav

Data_set1

<none>

Weighting <none>

Segment

N of rows in the file of

<none>

129

Syntax

CTABLES

/VLABELS VARIABLES=local category n_ulcera DISPLAY=LABEL

/TABLE categoria > local BY n_ulcera [COUNT F40.0, COLPCT.COUNT PCT40.1, TABLEPCT.COUNT PCT40.1]

/CATEGORIES

VARIABLES=local category ORDER=A KEY=VALUE EMPTY=INCLUDE TOTAL=YES POSITION=AFTER

MISSING=INCLUDE

/CATEGORIES

VARIABLES=n_ulcera ORDER=A KEY=VALUE EMPTY=EXCLUDE TOTAL=YES POSITION=AFTER.

Resources

Time

Time

00:00:00,09

00:00:00,12

|  | | | | n_HAPU | | | | | | | | | | | |
| --- | --- | --- | --- | --- | --- | --- | --- | --- | --- | --- | --- | --- | --- | --- | --- |
|  |  |  |  | 1 | | | 2 | | | 3 | | | Total | | |
|  |  |  |  | Counting | of column N | of table N | Counting | of column N | of table N | Counting | of column N | of table N | Counting | of column N | of table N |
| CATEGORY _1ª HAPU | CATEGORY 1 | LOCALIZATION _ 1ª HAPU | WIHTOUT UPP | 0 | 0,0% | 0,0% | 0 | 0,0% | 0,0% | 0 | 0,0% | 0,0% | 0 | 0,0% | 0,0% |
|  |  |  | occiput | 1 | 1,7% | ,8% | 0 | 0,0% | 0,0% | 0 | 0,0% | 0,0% | 1 | 1,4% | ,8% |
|  |  |  | shoulder blade | 1 | 1,7% | ,8% | 0 | 0,0% | 0,0% | 0 | 0,0% | 0,0% | 1 | 1,4% | ,8% |
|  |  |  | elbow | 2 | 3,4% | 1,6% | 1 | 9,1% | ,8% | 0 | 0,0% | 0,0% | 3 | 4,2% | 2,3% |
|  |  |  | sacralcoxis | 27 | 46,6% | 20,9% | 1 | 9,1% | ,8% | 1 | 50,0% | ,8% | 29 | 40,8% | 22,5% |
|  |  |  | heel | 19 | 32,8% | 14,7% | 6 | 54,5% | 4,7% | 1 | 50,0% | ,8% | 26 | 36,6% | 20,2% |
|  |  |  | toes | 0 | 0,0% | 0,0% | 0 | 0,0% | 0,0% | 0 | 0,0% | 0,0% | 0 | 0,0% | 0,0% |
|  |  |  | ear | 0 | 0,0% | 0,0% | 0 | 0,0% | 0,0% | 0 | 0,0% | 0,0% | 0 | 0,0% | 0,0% |
|  |  |  | shoulder | 0 | 0,0% | 0,0% | 0 | 0,0% | 0,0% | 0 | 0,0% | 0,0% | 0 | 0,0% | 0,0% |
|  |  |  | elbow | 0 | 0,0% | 0,0% | 0 | 0,0% | 0,0% | 0 | 0,0% | 0,0% | 0 | 0,0% | 0,0% |
|  |  |  | iliac crest | 0 | 0,0% | 0,0% | 0 | 0,0% | 0,0% | 0 | 0,0% | 0,0% | 0 | 0,0% | 0,0% |
|  |  |  | trochanter | 1 | 1,7% | ,8% | 0 | 0,0% | 0,0% | 0 | 0,0% | 0,0% | 1 | 1,4% | ,8% |
|  |  |  | knee (L.external) | 0 | 0,0% | 0,0% | 0 | 0,0% | 0,0% | 0 | 0,0% | 0,0% | 0 | 0,0% | 0,0% |
|  |  |  | knee (inner l.) | 0 | 0,0% | 0,0% | 0 | 0,0% | 0,0% | 0 | 0,0% | 0,0% | 0 | 0,0% | 0,0% |
|  |  |  | internal malleolus | 3 | 5,2% | 2,3% | 1 | 9,1% | ,8% | 0 | 0,0% | 0,0% | 4 | 5,6% | 3,1% |
|  |  |  | exterminal malleolus | 1 | 1,7% | ,8% | 1 | 9,1% | ,8% | 0 | 0,0% | 0,0% | 2 | 2,8% | 1,6% |
|  |  |  | elbow (p. ventral) | 0 | 0,0% | 0,0% | 0 | 0,0% | 0,0% | 0 | 0,0% | 0,0% | 0 | 0,0% | 0,0% |
|  |  |  | rib | 0 | 0,0% | 0,0% | 0 | 0,0% | 0,0% | 0 | 0,0% | 0,0% | 0 | 0,0% | 0,0% |
|  |  |  | front face of the thigh | 0 | 0,0% | 0,0% | 0 | 0,0% | 0,0% | 0 | 0,0% | 0,0% | 0 | 0,0% | 0,0% |
|  |  |  | knee (front) | 0 | 0,0% | 0,0% | 0 | 0,0% | 0,0% | 0 | 0,0% | 0,0% | 0 | 0,0% | 0,0% |
|  |  |  | toes (front) | 0 | 0,0% | 0,0% | 0 | 0,0% | 0,0% | 0 | 0,0% | 0,0% | 0 | 0,0% | 0,0% |
|  |  |  | other | 3 | 5,2% | 2,3% | 1 | 9,1% | ,8% | 0 | 0,0% | 0,0% | 4 | 5,6% | 3,1% |
|  |  |  | worthless | 0 | 0,0% | 0,0% | 0 | 0,0% | 0,0% | 0 | 0,0% | 0,0% | 0 | 0,0% | 0,0% |
|  |  |  | Total | 58 | 100,0% | 45,0% | 11 | 100,0% | 8,5% | 2 | 100,0% | 1,6% | 71 | 100,0% | 55,0% |
|  | CATEGORY 2 | LOCALIZATION_ 1ª HAPU | WIHTOUT UPP | 0 | 0,0% | 0,0% | 0 | 0,0% | 0,0% | 0 | 0,0% | 0,0% | 0 | 0,0% | 0,0% |
|  |  |  | occiput | 0 | 0,0% | 0,0% | 0 | 0,0% | 0,0% | 0 | 0,0% | 0,0% | 0 | 0,0% | 0,0% |
|  |  |  | shoulder blade | 0 | 0,0% | 0,0% | 0 | 0,0% | 0,0% | 0 | 0,0% | 0,0% | 0 | 0,0% | 0,0% |
|  |  |  | elbow | 2 | 3,9% | 1,6% | 0 | 0,0% | 0,0% | 0 | 0,0% | 0,0% | 2 | 3,6% | 1,6% |
|  |  |  | sacralcoxis | 28 | 54,9% | 21,7% | 2 | 50,0% | 1,6% | 0 | 0,0% | 0,0% | 30 | 54,5% | 23,3% |
|  |  |  | heel | 11 | 21,6% | 8,5% | 1 | 25,0% | ,8% | 0 | 0,0% | 0,0% | 12 | 21,8% | 9,3% |
|  |  |  | toes | 1 | 2,0% | ,8% | 0 | 0,0% | 0,0% | 0 | 0,0% | 0,0% | 1 | 1,8% | ,8% |
|  |  |  | ear | 0 | 0,0% | 0,0% | 0 | 0,0% | 0,0% | 0 | 0,0% | 0,0% | 0 | 0,0% | 0,0% |
|  |  |  | shoulder | 0 | 0,0% | 0,0% | 0 | 0,0% | 0,0% | 0 | 0,0% | 0,0% | 0 | 0,0% | 0,0% |
|  |  |  | elbow | 0 | 0,0% | 0,0% | 0 | 0,0% | 0,0% | 0 | 0,0% | 0,0% | 0 | 0,0% | 0,0% |
|  |  |  | iliac crest | 0 | 0,0% | 0,0% | 0 | 0,0% | 0,0% | 0 | 0,0% | 0,0% | 0 | 0,0% | 0,0% |
|  |  |  | trochanter | 1 | 2,0% | ,8% | 0 | 0,0% | 0,0% | 0 | 0,0% | 0,0% | 1 | 1,8% | ,8% |
|  |  |  | knee (L.external) | 0 | 0,0% | 0,0% | 0 | 0,0% | 0,0% | 0 | 0,0% | 0,0% | 0 | 0,0% | 0,0% |
|  |  |  | knee (inner l.) | 1 | 2,0% | ,8% | 0 | 0,0% | 0,0% | 0 | 0,0% | 0,0% | 1 | 1,8% | ,8% |
|  |  |  | internal malleolus | 2 | 3,9% | 1,6% | 0 | 0,0% | 0,0% | 0 | 0,0% | 0,0% | 2 | 3,6% | 1,6% |
|  |  |  | exterminal malleolus | 3 | 5,9% | 2,3% | 1 | 25,0% | ,8% | 0 | 0,0% | 0,0% | 4 | 7,3% | 3,1% |
|  |  |  | elbow (p. ventral) | 0 | 0,0% | 0,0% | 0 | 0,0% | 0,0% | 0 | 0,0% | 0,0% | 0 | 0,0% | 0,0% |
|  |  |  | rib | 0 | 0,0% | 0,0% | 0 | 0,0% | 0,0% | 0 | 0,0% | 0,0% | 0 | 0,0% | 0,0% |
|  |  |  | front face of the thigh | 0 | 0,0% | 0,0% | 0 | 0,0% | 0,0% | 0 | 0,0% | 0,0% | 0 | 0,0% | 0,0% |
|  |  |  | knee (front) | 0 | 0,0% | 0,0% | 0 | 0,0% | 0,0% | 0 | 0,0% | 0,0% | 0 | 0,0% | 0,0% |
|  |  |  | toes (front) | 0 | 0,0% | 0,0% | 0 | 0,0% | 0,0% | 0 | 0,0% | 0,0% | 0 | 0,0% | 0,0% |
|  |  |  | other | 2 | 3,9% | 1,6% | 0 | 0,0% | 0,0% | 0 | 0,0% | 0,0% | 2 | 3,6% | 1,6% |
|  |  |  | worthless | 0 | 0,0% | 0,0% | 0 | 0,0% | 0,0% | 0 | 0,0% | 0,0% | 0 | 0,0% | 0,0% |
|  |  |  | Total | 51 | 100,0% | 39,5% | 4 | 100,0% | 3,1% | 0 | 0,0% | 0,0% | 55 | 100,0% | 42,6% |
|  | CATEGORY 3 | LOCALIZATION _ 1ª HAPU | WIHTOUT UPP | 0 | 0,0% | 0,0% | 0 | 0,0% | 0,0% | 0 | 0,0% | 0,0% | 0 | 0,0% | 0,0% |
|  |  |  | occiput | 0 | 0,0% | 0,0% | 0 | 0,0% | 0,0% | 0 | 0,0% | 0,0% | 0 | 0,0% | 0,0% |
|  |  |  | shoulder blade | 0 | 0,0% | 0,0% | 0 | 0,0% | 0,0% | 0 | 0,0% | 0,0% | 0 | 0,0% | 0,0% |
|  |  |  | elbow | 0 | 0,0% | 0,0% | 0 | 0,0% | 0,0% | 0 | 0,0% | 0,0% | 0 | 0,0% | 0,0% |
|  |  |  | sacralcoxis | 2 | 66,7% | 1,6% | 0 | 0,0% | 0,0% | 0 | 0,0% | 0,0% | 2 | 66,7% | 1,6% |
|  |  |  | heel | 1 | 33,3% | ,8% | 0 | 0,0% | 0,0% | 0 | 0,0% | 0,0% | 1 | 33,3% | ,8% |
|  |  |  | toes | 0 | 0,0% | 0,0% | 0 | 0,0% | 0,0% | 0 | 0,0% | 0,0% | 0 | 0,0% | 0,0% |
|  |  |  | ear | 0 | 0,0% | 0,0% | 0 | 0,0% | 0,0% | 0 | 0,0% | 0,0% | 0 | 0,0% | 0,0% |
|  |  |  | shoulder | 0 | 0,0% | 0,0% | 0 | 0,0% | 0,0% | 0 | 0,0% | 0,0% | 0 | 0,0% | 0,0% |
|  |  |  | elbow | 0 | 0,0% | 0,0% | 0 | 0,0% | 0,0% | 0 | 0,0% | 0,0% | 0 | 0,0% | 0,0% |
|  |  |  | iliac crest | 0 | 0,0% | 0,0% | 0 | 0,0% | 0,0% | 0 | 0,0% | 0,0% | 0 | 0,0% | 0,0% |
|  |  |  | trochanter | 0 | 0,0% | 0,0% | 0 | 0,0% | 0,0% | 0 | 0,0% | 0,0% | 0 | 0,0% | 0,0% |
|  |  |  | knee (L.external) | 0 | 0,0% | 0,0% | 0 | 0,0% | 0,0% | 0 | 0,0% | 0,0% | 0 | 0,0% | 0,0% |
|  |  |  | knee (inner l.) | 0 | 0,0% | 0,0% | 0 | 0,0% | 0,0% | 0 | 0,0% | 0,0% | 0 | 0,0% | 0,0% |
|  |  |  | internal malleolus | 0 | 0,0% | 0,0% | 0 | 0,0% | 0,0% | 0 | 0,0% | 0,0% | 0 | 0,0% | 0,0% |
|  |  |  | exterminal malleolus | 0 | 0,0% | 0,0% | 0 | 0,0% | 0,0% | 0 | 0,0% | 0,0% | 0 | 0,0% | 0,0% |
|  |  |  | elbow (p. ventral) | 0 | 0,0% | 0,0% | 0 | 0,0% | 0,0% | 0 | 0,0% | 0,0% | 0 | 0,0% | 0,0% |
|  |  |  | rib | 0 | 0,0% | 0,0% | 0 | 0,0% | 0,0% | 0 | 0,0% | 0,0% | 0 | 0,0% | 0,0% |
|  |  |  | front face of the thigh | 0 | 0,0% | 0,0% | 0 | 0,0% | 0,0% | 0 | 0,0% | 0,0% | 0 | 0,0% | 0,0% |
|  |  |  | knee (front) | 0 | 0,0% | 0,0% | 0 | 0,0% | 0,0% | 0 | 0,0% | 0,0% | 0 | 0,0% | 0,0% |
|  |  |  | toes (front) | 0 | 0,0% | 0,0% | 0 | 0,0% | 0,0% | 0 | 0,0% | 0,0% | 0 | 0,0% | 0,0% |
|  |  |  | other | 0 | 0,0% | 0,0% | 0 | 0,0% | 0,0% | 0 | 0,0% | 0,0% | 0 | 0,0% | 0,0% |
|  |  |  | worthless | 0 | 0,0% | 0,0% | 0 | 0,0% | 0,0% | 0 | 0,0% | 0,0% | 0 | 0,0% | 0,0% |
|  |  |  | Total | 3 | 100,0% | 2,3% | 0 | 0,0% | 0,0% | 0 | 0,0% | 0,0% | 3 | 100,0% | 2,3% |
|  | Total | LOCALIZATION _ 1ª HAPU | WIHTOUT HAPU | 0 | 0,0% | 0,0% | 0 | 0,0% | 0,0% | 0 | 0,0% | 0,0% | 0 | 0,0% | 0,0% |
|  |  |  | occiput | 1 | ,9% | ,8% | 0 | 0,0% | 0,0% | 0 | 0,0% | 0,0% | 1 | ,8% | ,8% |
|  |  |  | shoulder blade | 1 | ,9% | ,8% | 0 | 0,0% | 0,0% | 0 | 0,0% | 0,0% | 1 | ,8% | ,8% |
|  |  |  | elbow | 4 | 3,6% | 3,1% | 1 | 6,7% | ,8% | 0 | 0,0% | 0,0% | 5 | 3,9% | 3,9% |
|  |  |  | sacralcoxis | 57 | 50,9% | 44,2% | 3 | 20,0% | 2,3% | 1 | 50,0% | ,8% | 61 | 47,3% | 47,3% |
|  |  |  | heel | 31 | 27,7% | 24,0% | 7 | 46,7% | 5,4% | 1 | 50,0% | ,8% | 39 | 30,2% | 30,2% |
|  |  |  | toes | 1 | ,9% | ,8% | 0 | 0,0% | 0,0% | 0 | 0,0% | 0,0% | 1 | ,8% | ,8% |
|  |  |  | ear | 0 | 0,0% | 0,0% | 0 | 0,0% | 0,0% | 0 | 0,0% | 0,0% | 0 | 0,0% | 0,0% |
|  |  |  | shoulder | 0 | 0,0% | 0,0% | 0 | 0,0% | 0,0% | 0 | 0,0% | 0,0% | 0 | 0,0% | 0,0% |
|  |  |  | elbow | 0 | 0,0% | 0,0% | 0 | 0,0% | 0,0% | 0 | 0,0% | 0,0% | 0 | 0,0% | 0,0% |
|  |  |  | iliac crest | 0 | 0,0% | 0,0% | 0 | 0,0% | 0,0% | 0 | 0,0% | 0,0% | 0 | 0,0% | 0,0% |
|  |  |  | trochanter | 2 | 1,8% | 1,6% | 0 | 0,0% | 0,0% | 0 | 0,0% | 0,0% | 2 | 1,6% | 1,6% |
|  |  |  | knee (L.external) | 0 | 0,0% | 0,0% | 0 | 0,0% | 0,0% | 0 | 0,0% | 0,0% | 0 | 0,0% | 0,0% |
|  |  |  | knee (inner l.) | 1 | ,9% | ,8% | 0 | 0,0% | 0,0% | 0 | 0,0% | 0,0% | 1 | ,8% | ,8% |
|  |  |  | internal malleolus | 5 | 4,5% | 3,9% | 1 | 6,7% | ,8% | 0 | 0,0% | 0,0% | 6 | 4,7% | 4,7% |
|  |  |  | exterminal malleolus | 4 | 3,6% | 3,1% | 2 | 13,3% | 1,6% | 0 | 0,0% | 0,0% | 6 | 4,7% | 4,7% |
|  |  |  | elbow (p. ventral) | 0 | 0,0% | 0,0% | 0 | 0,0% | 0,0% | 0 | 0,0% | 0,0% | 0 | 0,0% | 0,0% |
|  |  |  | rib | 0 | 0,0% | 0,0% | 0 | 0,0% | 0,0% | 0 | 0,0% | 0,0% | 0 | 0,0% | 0,0% |
|  |  |  | front face of the thigh | 0 | 0,0% | 0,0% | 0 | 0,0% | 0,0% | 0 | 0,0% | 0,0% | 0 | 0,0% | 0,0% |
|  |  |  | knee (front) | 0 | 0,0% | 0,0% | 0 | 0,0% | 0,0% | 0 | 0,0% | 0,0% | 0 | 0,0% | 0,0% |
|  |  |  | toes (front) | 0 | 0,0% | 0,0% | 0 | 0,0% | 0,0% | 0 | 0,0% | 0,0% | 0 | 0,0% | 0,0% |
|  |  |  | other | 5 | 4,5% | 3,9% | 1 | 6,7% | ,8% | 0 | 0,0% | 0,0% | 6 | 4,7% | 4,7% |
|  |  |  | worthless | 0 | 0,0% | 0,0% | 0 | 0,0% | 0,0% | 0 | 0,0% | 0,0% | 0 | 0,0% | 0,0% |
|  |  |  | Total | 112 | 100,0% | 86,8% | 15 | 100,0% | 11,6% | 2 | 100,0% | 1,6% | 129 | 100,0% | 100,0% |

Notes

| Output created |  | 01-MAY-2019 16:04:46 |
| --- | --- | --- |
| Comments |  |  |
| Entrance | Facts | C: Users. Diaz\Desktop\ELIA 1 5 2019\BBDD 1260.sav |
|  | Set of | Data_set2 |
|  | Filter | <none> |
|  | Weighting | <none> |
|  | Segment | <none> |
|  | N of rows in the file of | 1260 |
| Management of lost value | Definition of absence | User-defined missing values are treated as lost. |
|  | Cases used | The statistics are based on all cases with valid data. |
| Syntax |  | FREQUENCIES VARIABLES=SEX AGRUP_AGRUP_AGRUP UNIT_REGISTRO_c DXM FACT_RIESGO_AGRUPADO_2 ALT_DE_LA_MOVILIDAD EXCESO_PRESION ALT_NIVEL_CONCIENCIA ALT_ACTIVITY ALT_NUTRITION ALT_TEMPERATURE DET_SENSIBILI_CUTANEA EFFECT_SECUN_TTº INCONTINENCE ADVANCED_AGE DAYS_OF_INGR_GROUP_2  /ORDER=ANALYSIS. |
| Resources | Time | 00:00:00,03 |
|  | Time | 00:00:00,03 |

# Frequency Table

GENDER

|  | | Frequency | Percentage | Valid percentage | Cumulative percentage |
| --- | --- | --- | --- | --- | --- |
| Valid | MALE | 598 | 47,5 | 47,5 | 47,5 |
|  | FEMALE | 662 | 52,5 | 52,5 | 100,0 |
|  | Total | 1260 | 100,0 | 100,0 |  |

HOSPITALLIZATION_WARDS

|  | | Frequency | Percentage | Valid percentage | Cumulative percentage |
| --- | --- | --- | --- | --- | --- |
| Valid | SURGICAL | 239 | 19,0 | 19,0 | 19,0 |
|  | MEDICAL- | 570 | 45,2 | 45,2 | 64,2 |
|  | MEDICAL- SURGICAL | 451 | 35,8 | 35,8 | 100,0 |
|  | Total | 1260 | 100,0 | 100,0 |  |

PRINCIPAL_DIAGNOSTIC_MEDICAL_ REASON FOR ADMISSION

|  | | Frequency | Percentage | Valid percentage | Cumulative percentage |
| --- | --- | --- | --- | --- | --- |
| Valid | CARDIOVASC | 252 | 20,0 | 20,0 | 20,0 |
|  | RESPIRATORI | 205 | 16,3 | 16,3 | 36,3 |
|  | URINARY | 85 | 6,7 | 6,7 | 43,0 |
|  | NERVOUS SYSTEM | 99 | 7,9 | 7,9 | 50,9 |
|  | ENDOCRINE/D IABETES | 20 | 1,6 | 1,6 | 52,5 |
|  | ONCOLOGY | 69 | 5,5 | 5,5 | 57,9 |
|  | SEPSIS | 81 | 6,4 | 6,4 | 64,4 |
|  | DIGESTIVE | 180 | 14,3 | 14,3 | 78,7 |
|  | TRAUMATOLO | 262 | 20,8 | 20,8 | 99,4 |
|  | ORL | 7 | ,6 | ,6 | 100,0 |
|  | Total | 1260 | 100,0 | 100,0 |  |

ALTERATION_ MOBILITY

|  | | Frequency | Percentage | Valid percentage | Cumulative percentage |
| --- | --- | --- | --- | --- | --- |
| Valid | NO | 361 | 28,7 | 28,7 | 28,7 |
|  | YES | 899 | 71,3 | 71,3 | 100,0 |
|  | Total | 1260 | 100,0 | 100,0 |  |

EXCESS_PRESSURE

|  | | Frequency | Percentage | Valid percentage | Cumulative percentage |
| --- | --- | --- | --- | --- | --- |
| Valid | NO | 831 | 66,0 | 66,0 | 66,0 |
|  | YES | 429 | 34,0 | 34,0 | 100,0 |
|  | Total | 1260 | 100,0 | 100,0 |  |

ALTERATION_LEVEL_CONSCIOUSNESS

|  | | Frequency | Percentage | Valid percentage | Cumulative percentage |
| --- | --- | --- | --- | --- | --- |
| Valid | NO | 1155 | 91,7 | 91,7 | 91,7 |
|  | YES | 105 | 8,3 | 8,3 | 100,0 |
|  | Total | 1260 | 100,0 | 100,0 |  |

ALTALTERATION_ ACTIVITY

|  | | Frequency | Percentage | Valid percentage | Cumulative percentage |
| --- | --- | --- | --- | --- | --- |
| Valid | NO | 408 | 32,4 | 32,4 | 32,4 |
|  | YES | 852 | 67,6 | 67,6 | 100,0 |
|  | Total | 1260 | 100,0 | 100,0 |  |

ALTERATION_NUTRITION

|  | | Frequency | Percentage | Valid percentage | Cumulative percentage |
| --- | --- | --- | --- | --- | --- |
| Valid | NO | 532 | 42,2 | 42,2 | 42,2 |
|  | YES | 728 | 57,8 | 57,8 | 100,0 |
|  | Total | 1260 | 100,0 | 100,0 |  |

ALTERATION_BODY_TEMPERATURE

|  | | Frequency | Percentage | Valid percentage | Cumulative percentage |
| --- | --- | --- | --- | --- | --- |
| Valid | NO | 1003 | 79,6 | 79,6 | 79,6 |
|  | YES | 257 | 20,4 | 20,4 | 100,0 |
|  | Total | 1260 | 100,0 | 100,0 |  |

ALTERATION_SKIN_SENSITIBITY

|  | | Frequency | Percentage | Valid percentage | Cumulative percentage |
| --- | --- | --- | --- | --- | --- |
| Valid | NO | 789 | 62,6 | 62,6 | 62,6 |
|  | YES | 471 | 37,4 | 37,4 | 100,0 |
|  | Total | 1260 | 100,0 | 100,0 |  |

SECONDARY_EFFECTS_OF_ TTº_MEDICAL

|  | | Frequency | Percentage | Valid percentage | Cumulative percentage |
| --- | --- | --- | --- | --- | --- |
| Valid | NO | 706 | 56,0 | 56,0 | 56,0 |
|  | YES | 554 | 44,0 | 44,0 | 100,0 |
|  | Total | 1260 | 100,0 | 100,0 |  |

INCONTINENCE

|  | | Frequency | Percentage | Valid percentage | Cumulative percentage |
| --- | --- | --- | --- | --- | --- |
| Valid | NO | 790 | 62,7 | 62,7 | 62,7 |
|  | YES | 470 | 37,3 | 37,3 | 100,0 |
|  | Total | 1260 | 100,0 | 100,0 |  |

AGE_> 65_YEARS

|  | | Frequency | Percentage | Valid percentage | Cumulative percentage |
| --- | --- | --- | --- | --- | --- |
| Valid | NO | 219 | 17,4 | 17,4 | 17,4 |
|  | YES | 1041 | 82,6 | 82,6 | 100,0 |
|  | Total | 1260 | 100,0 | 100,0 |  |

## VARIABLE FREQUENCIES=EDAD NORTON score INCOME_DAYS

/FORMAT=NOTABLE

/NTILES=4

/STATISTICS=STDDEV MINIMUM MAXIMUM MEAN

/ORDER=ANALYSIS.

**Frequencies**

Notes

| Output created |  | 01-MAY-2019 16:05:03  C: Users. Diaz\Desktop\ELIA 1 5 2019\BBDD 1260.sav  Data_set2  <none>  <none>  <none>  1260  User-defined missing values are treated as lost.  The statistics are based on all cases with valid data.  FREQUENCIES VARIABLES=AGE  NORTON score INCOME_DAYS  /FORMAT=NOTABLE  /NTILES=4  /STATISTICS=STDDEV MINIMUM MAXIMUM MEAN  /ORDER=ANALYSIS.  00:00:00,02  00:00:00,05 |
| --- | --- | --- |
| Comments |  |  |
| Entrance | Facts |  |
|  | Set of |  |
|  | Filter |  |
|  | Weighting |  |
|  | Segment |  |
|  | N of rows in the |  |
|  | file of |  |
| Management of lost value | Definition |  |
|  | absence |  |
|  | Cases |  |
|  | used |  |
| Syntax |  |  |
| Resources | Time |  |
|  | Time |  |

Statistics

|  | | PATIENT`S AGE | SCORE SCALE NORTON-MI | TOTAL DAYS OF INCOME |
| --- | --- | --- | --- | --- |
| N | Valid | 1260 | 1260 | 1260 |
|  | Lost | 0 | 0 | 0 |
| Medium |  | 76,07 | 16,87 | 11,61 |
| Standard deviation |  | 14,341 | 1,646 | 11,129 |
| Minimum |  | 19 | 15 | 1 |
| Maximum |  | 102 | 20 | 140 |
| Percentiles | 25 | 69,25 | 15,00 | 6,00 |
|  | 50 | 80,00 | 16,00 | 9,00 |
|  | 75 | 86,00 | 18,00 | 14,00 |

## CROSSTABS

/TABLES=SEX

AGRUP_AGRUP UNIT_REGISTRO_c DXM FACT_RIESGO_AGRUPADO_2 ALT_DE_LA_MOVILIDAD EXCESO_PRESION ALT_NIVEL_CONCIENCIA ALT_ACTIVIDAD

ALT_NUTRITION ALT_TEMPERATURE DET_SENSIBILI_CUTANEA EFFECT_SECUN_TTº INCONTINENCE ADVANCED_AGE DAYS_OF_INGR_GROUP_2

BY PATIENTS_WITH_UPP_INTRA

/FORMAT=AVALUE TABLES

/CELLS=COUNT COLUMN

/COUNT ROUND CELL.

**Cross tables**

Notes

| Output created |  | 01-MAY-2019 16:05:12 |
| --- | --- | --- |
| Comments |  |  |
| Entrance | Facts | C: Users. Diaz\Desktop\ELIA 1 5 2019\BBDD 1260.sav |
|  | Set of | Data_set2 |
|  | Filter | <none> |
|  | Weighting | <none> |
|  | Segment | <none> |
|  | N of rows in the file of | 1260 |
| Management of lost value | Definition of absence | User-defined missing values are treated as lost. |
|  | Cases used | The statistics for each table are based on all cases with valid data in the ranges specified for all variables in each table. |
| Syntax |  | CROSSTABS  /FACT_RIESGO_AGRUPADO_2 ALT_DE_LA_MOVILIDAD EXCESO_PRESION ALT_CONCIENCIA_Level_CONCIENCIA ALT_ACTIVITY ALT_NUTRITION ALT_TEMPERATURE DET_SENSIBILI_CUTANEA EFFECT_SECUN_TTº INCONTINENCE ADVANCED_AGE DAYS_OF_INGR_GROUP_2  BY PATIENTS_WITH_UPP_INTRA  /FORMAT=AVALUE TABLES  /CELLS=COUNT COLUMN  /COUNT ROUND CELL. |
| Resources | Time | 00:00:00,03 |
|  | Time | 00:00:00,05 |
|  | Dimensions | 2 |
|  | Boxes | 131029 |

**GENDER*PACENT IN WITH PROTOCOL UPP INTRA cross tabulation**

|  | | | PATIENTS | | Total |
| --- | --- | --- | --- | --- | --- |
|  |  |  | WITH HAPU | WITHOUT  HAPU |  |
| GENDER | MALE | Counting | 60 | 538 | 598 |
|  |  | within PATIENTS IN WITH PROTOCOL UPP INTRA | 53,6% | 46,9% | 47,5% |
|  | FEMALE | Counting | 52 | 610 | 662 |
|  |  | within PATIENTS IN WITH PROTOCOL UPP INTRA | 46,4% | 53,1% | 52,5% |
| Total |  | Counting | 112 | 1148 | 1260 |
|  |  | within PATIENTS IN WITH PROTOCOL UPP INTRA | 100,0% | 100,0% | 100,0% |

HOSPITALIZATION_WARDS*PACIENT WITH PROTOCOL UPP INTRA cross tabulation

|  | | | PATIENTS | | Total |
| --- | --- | --- | --- | --- | --- |
|  |  |  | WITH HAPU | WITHOUT HAPU |  |
| Hospitalization_Wards | Surgical | Counting | 23 | 216 | 239 |
|  |  | within PATIENTS IN WITH PROTOCOL UPP INTRA | 20,5% | 18,8% | 19,0% |
|  | Medical | Counting | 58 | 512 | 570 |
|  |  | within PATIENTS IN WITH PROTOCOL UPP INTRA | 51,8% | 44,6% | 45,2% |
|  | Medical- Surgical | Counting | 31 | 420 | 451 |
|  |  | within PATIENTS IN WITH PROTOCOL UPP INTRA | 27,7% | 36,6% | 35,8% |
| Total |  | Counting | 112 | 1148 | 1260 |
|  |  | within PATIENTS IN WITH PROTOCOL UPP INTRA | 100,0% | 100,0% | 100,0% |

PRINCIPAL_DIAGNOSTIC_ MEDICAL*PACENT IN WITH PROTOCOL UPP INTRA cross tabulation

|  | | | PATIENTS | | Total |
| --- | --- | --- | --- | --- | --- |
|  |  |  | WITH HAPU | WITHOUT HAPU |  |
| MAIN_DIAGNOSTI C_MEDICAL | CARDIOVASC ULAR | Counting | 15 | 237 | 252 |
|  |  | within PATIENTS IN PROTOCOL UPP INTRA | 13,4% | 20,6% | 20,0% |
|  | RESPIRATOR AND | Counting | 29 | 176 | 205 |
|  |  | within PATIENTS IN PROTOCOL UPP INTRA | 25,9% | 15,3% | 16,3% |
|  | URINARY | Counting | 8 | 77 | 85 |
|  |  | within PATIENTS IN PROTOCOL UPP INTRA | 7,1% | 6,7% | 6,7% |
|  | NERVOUS_SY STEM | Counting | 6 | 93 | 99 |
|  |  | within PATIENTS IN PROTOCOL UPP INTRA | 5,4% | 8,1% | 7,9% |
|  | ENDOCRINE/D IABETES | Counting | 4 | 16 | 20 |
|  |  | within PATIENTS IN PROTOCOL UPP INTRA | 3,6% | 1,4% | 1,6% |
|  | ONCOLOGY | Counting | 7 | 62 | 69 |
|  |  | within PATIENTS IN PROTOCOL UPP INTRA | 6,3% | 5,4% | 5,5% |
|  | SEPSIS | Counting | 9 | 72 | 81 |
|  |  | within PATIENTS IN PROTOCOL UPP INTRA | 8,0% | 6,3% | 6,4% |
|  | DIGESTIVE | Counting | 15 | 165 | 180 |
|  |  | within PATIENTS IN PROTOCOL UPP INTRA | 13,4% | 14,4% | 14,3% |
|  | TRAUMATOLO GY | Counting | 18 | 244 | 262 |
|  |  | within PATIENTS IN PROTOCOL UPP INTRA | 16,1% | 21,3% | 20,8% |
|  | ORL | Counting | 1 | 6 | 7 |
|  |  | within PATIENTS IN PROTOCOL UPP INTRA | ,9% | 6 | ,6% |
| Total |  | Counting | 112 | 1148 | 1260 |
|  |  | within PATIENTS IN PROTOCOL UPP INTRA | 100,0% | 100,0% | 100,0% |

RISK_FACTORS_AGRUPADO_2*PACIENT IN WITH PROTOCOL UPP INTRA cross tabulation

|  | | | PATIENTS | | Total |
| --- | --- | --- | --- | --- | --- |
|  |  |  | WITH HAPU | WITHOUT  HAPU |  |
| RISK_FACTORS_GROUPED_2 | 0 – 2 | Counting | 23 | 233 | 256 |
|  |  | within PATIENTS IN PROTOCOL UPP INTRA | 20,5% | 20,3% | 20,3% |
|  | 3 – 4 | Counting | 33 | 293 | 326 |
|  |  | within PATIENTS IN PROTOCOL UPP INTRA | 29,5% | 25,5% | 25,9% |
|  | 5 – 6 | Counting | 38 | 385 | 423 |
|  |  | within PATIENTS IN PROTOCOL UPP INTRA | 33,9% | 33,5% | 33,6% |
|  | 7 – 8 | Counting | 17 | 165 | 182 |
|  |  | within PATIENTS IN PROTOCOL UPP INTRA | 15,2% | 14,4% | 14,4% |
|  | 9 – 10 | Counting | 1 | 72 | 73 |
|  |  | within PATIENTS IN PROTOCOL UPP INTRA | ,9% | 6,3% | 5,8% |
| Total |  | Counting | 112 | 1148 | 1260 |
|  |  | within PATIENTS IN PROTOCOL UPP INTRA | 100,0% | 100,0% | 100,0% |

ALTERATION_ MOBILITY *PACIENTS WITH PROTOCOL UPP INTRA cross tabulation

|  | | | PATIENTS | | Total |
| --- | --- | --- | --- | --- | --- |
|  |  |  | WITH HAPU | WITHOUT HAPU |  |
| ALTERATION_MOBILITY | NO | Counting | 40 | 321 | 361 |
|  |  | within PATIENTS IN PROTOCOL UPP INTRA | 35,7% | 28,0% | 28,7% |
|  | YES | Counting | 72 | 827 | 899 |
|  |  | within PATIENTS IN PROTOCOL UPP INTRA | 64,3% | 72,0% | 71,3% |
| Total |  | Counting | 112 | 1148 | 1260 |
|  |  | within PATIENTS IN PROTOCOL UPP INTRA | 100,0% | 100,0% | 100,0% |

EXCESS_PRESSURE*PACIENT WITH PROTOCOL UPP INTRA cross tabulation

|  | | | PATIENTS | | Total |
| --- | --- | --- | --- | --- | --- |
|  |  |  | WITH HAPU | WITHOUT HAPU |  |
| EXCESS_PRESSURE | NO | Counting | 42 | 789 | 831 |
|  |  | within PATIENTS IN PROTOCOL UPP INTRA | 37,5% | 68,7% | 66,0% |
|  | YES | Counting | 70 | 359 | 429 |
|  |  | within PATIENTS IN PROTOCOL UPP INTRA | 62,5% | 31,3% | 34,0% |
| Total |  | Counting | 112 | 1148 | 1260 |
|  |  | within PATIENTS IN PROTOCOL UPP INTRA | 100,0% | 100,0% | 100,0% |

ALTERATION_LEVEL_OF_CONSCIOUSNESS*PACIENTS WITH PROTOCOL UPP INTRA cross tabulation

|  | | | PATIENTS | | Total |
| --- | --- | --- | --- | --- | --- |
|  |  |  | WITH HAPU | WITHOUT HAPU |  |
| ALTERATION_LEVEL_O F_CONSCIOUSNESS | NO | Counting | 102 | 1053 | 1155 |
|  |  | within PATIENTS IN PROTOCOL UPP INTRA | 91,1% | 91,7% | 91,7% |
|  | YES | Counting | 10 | 95 | 105 |
|  |  | within PATIENTS IN PROTOCOL UPP INTRA | 8,9% | 8,3% | 8,3% |
| Total |  | Counting | 112 | 1148 | 1260 |
|  |  | within PATIENTS IN PROTOCOL UPP INTRA | 100,0% | 100,0% | 100,0% |

ALTALTERATION_ACTIVITY*PACIENTS WITH PROTOCOL UPP INTRA cross tabulation

|  | | | PATIENTS | | Total |
| --- | --- | --- | --- | --- | --- |
|  |  |  | WITH HAPU | WITHOUT HAPU |  |
| ALTALTERATION_ACTIVI TY | NO | Counting | 30 | 378 | 408 |
|  |  | within PATIENTS IN PROTOCOL UPP INTRA | 26,8% | 32,9% | 32,4% |
|  | YES | Counting | 82 | 770 | 852 |
|  |  | within PATIENTS IN PROTOCOL UPP INTRA | 73,2% | 67,1% | 67,6% |
| Total |  | Counting | 112 | 1148 | 1260 |
|  |  | within PATIENTS IN PROTOCOL UPP INTRA | 100,0% | 100,0% | 100,0% |

ALTERATION_NUTRITION*PACENT WITH PROTOCOL UPP INTRA cross tabulation

|  | | | PATIENTS | | Total |
| --- | --- | --- | --- | --- | --- |
|  |  |  | WITH HAPU | WITHOUT HAPU |  |
| ALTERATION_NUTRITION | NO | Counting | 79 | 453 | 532 |
|  |  | within PATIENTS IN PROTOCOL UPP INTRA | 70,5% | 39,5% | 42,2% |
|  | YES | Counting | 33 | 695 | 728 |
|  |  | within PATIENTS IN PROTOCOL UPP INTRA | 29,5% | 60,5% | 57,8% |
| Total |  | Counting | 112 | 1148 | 1260 |
|  |  | within PATIENTS IN PROTOCOL UPP INTRA | 100,0% | 100,0% | 100,0% |

ALTERATION_BODY_TEMPERATURE *PACIENT WITH PROTOCOL UPP INTRA cross tabulation

|  | | | PATIENTS | | Total |
| --- | --- | --- | --- | --- | --- |
|  |  |  | WITH HAPU | WITHOUT HAPU |  |
| ALTERATION_BODY_TE MPERATURE | NO | Counting | 84 | 919 | 1003 |
|  |  | within PATIENTS IN PROTOCOL UPP INTRA | 75,0% | 80,1% | 79,6% |
|  | YES | Counting | 28 | 229 | 257 |
|  |  | within PATIENTS IN PROTOCOL UPP INTRA | 25,0% | 19,9% | 20,4% |
| Total |  | Counting | 112 | 1148 | 1260 |
|  |  | within PATIENTS IN PROTOCOL UPP INTRA | 100,0% | 100,0% | 100,0% |

ALTERATION_SKIN_SENSITIVITY*PACIENTS WITH PROTOCOL UPP INTRA cross tabulation

|  | | | PATIENTS | | Total |
| --- | --- | --- | --- | --- | --- |
|  |  |  | WITH HAPU | WITHOUT HAPU |  |
| ALTERATION_SKIN_SEN SITIVITY | NO | Counting | 58 | 731 | 789 |
|  |  | within PATIENTS IN PROTOCOL UPP INTRA | 51,8% | 63,7% | 62,6% |
|  | YES | Counting | 54 | 417 | 471 |
|  |  | within PATIENTS IN PROTOCOL UPP INTRA | 48,2% | 36,3% | 37,4% |
| Total |  | Counting | 112 | 1148 | 1260 |
|  |  | within PATIENTS IN PROTOCOL UPP INTRA | 100,0% | 100,0% | 100,0% |

SECONDARY_EFFECTS_OF_TTº_MEDICAL*PACENT IN PROTOCOL UPP INTRA cross tabulation

|  | | | PATIENTS | | Total |
| --- | --- | --- | --- | --- | --- |
|  |  |  | WITH HAPU | WITHOUT  HAPU |  |
| SECUNDARY_EFFECTS  _OF_TTº_MEDICAL | NO | Counting | 92 | 614 | 706 |
|  |  | within PATIENTS IN PROTOCOL UPP INTRA | 82,1% | 53,5% | 56,0% |
|  | YES | Counting | 20 | 534 | 554 |
|  |  | within PATIENTS IN PROTOCOL UPP INTRA | 17,9% | 46,5% | 44,0% |
| Total |  | Counting | 112 | 1148 | 1260 |
|  |  | within PATIENTS IN PROTOCOL UPP INTRA | 100,0% | 100,0% | 100,0% |

INCONTINENCE*PACENT WITH PROTOCOL UPP INTRA cross tabulation

|  | | | PATIENTS | | Total |
| --- | --- | --- | --- | --- | --- |
|  |  |  | WITH HAPU | WITHOUT HAPU |  |
| INCONTINENCE | NO | Counting | 82 | 708 | 790 |
|  |  | within PATIENTS IN PROTOCOL UPP INTRA | 73,2% | 61,7% | 62,7% |
|  | YES | Counting | 30 | 440 | 470 |
|  |  | within PATIENTS IN PROTOCOL UPP INTRA | 26,8% | 38,3% | 37,3% |
| Total |  | Counting | 112 | 1148 | 1260 |
|  |  | within PATIENTS IN PROTOCOL UPP INTRA | 100,0% | 100,0% | 100,0% |

AGE > 65_YEARS *PACENT IN PROTOCOL UPP INTRA cross tabulation

|  | | | PATIENTS | | Total |
| --- | --- | --- | --- | --- | --- |
|  |  |  | WITH HAPU | WITHOUT HAPU |  |
| AGE > 65 YEARS | NO | Counting | 15 | 204 | 219 |
|  |  | within PATIENTS IN PROTOCOL UPP INTRA | 13,4% | 17,8% | 17,4% |
|  | YES | Counting | 97 | 944 | 1041 |
|  |  | within PATIENTS IN PROTOCOL UPP INTRA | 86,6% | 82,2% | 82,6% |
| Total |  | Counting | 112 | 1148 | 1260 |
|  |  | within PATIENTS IN PROTOCOL UPP INTRA | 100,0% | 100,0% | 100,0% |

## SORT CASES BY PACIENTES_CON_UPP_INTRA.

SPLIT FILE LAYERED BY PACIENTES_CON_UPP_INTRA.

VARIABLE FREQUENCIES=EDAD NORTON score INCOME_DAYS

/FORMAT=NOTABLE

/NTILES=4

/STATISTICS=STDDEV MINIMUM MAXIMUM MEAN

/ORDER=ANALYSIS.

**Frequencies**

Notes

| Output created |  | 01-MAY-2019 16:05:26 |
| --- | --- | --- |
| Comments |  |  |
| Entrance | Facts | C: Users. Diaz\Desktop\ELIA 1 5 2019\BBDD 1260.sav |
|  | Set of | Data_set2 |
|  | Filter | <none> |
|  | Weighting | <none> |
|  | Segment  archive | PATIENTS WITH INTRA-UPP PROTOCOL |
|  | N of rows in the file of | 1260 |
| Management of lost value | Definition  absence | User-defined missing values are treated as lost. |
|  | Cases used | The statistics are based on all cases with valid data. |
| Syntax | Time | FREQUENCIES VARIABLES=AGE  NORTON score INCOME_DAYS  /FORMAT=NOTABLE  /NTILES=4  /STATISTICS=STDDEV MINIMUM MAXIMUM MEAN  /ORDER=ANALYSIS. |
| Resources |  | 00:00:00,00 |
|  | Time | 00:00:00,02 |

Statistics

| PATIENTS IN HAPU PROTOCOL | | | PATIENT`S AGE | SCORE ON THE NORTON-MI SCALE | DAYS TOTAL OF HOSPITALIZATIO N |
| --- | --- | --- | --- | --- | --- |
| WIHT HAPU | N | Valid | 112 | 112 | 112 |
|  |  | Lost | 0 | 0 | 0 |
|  | Medium | | 76,81 | 16,25 | 21,26 |
|  | Standard deviation | | 11,750 | 1,504 | 18,702 |
|  | Minimum | | 49 | 15 | 2 |
|  | Maximum | | 98 | 20 | 113 |
|  | Percentiles | 25 | 69,25 | 15,00 | 9,00 |
|  |  | 50 | 79,50 | 16,00 | 15,00 |
|  |  | 75 | 86,00 | 17,00 | 28,75 |
| WIHTOUT HAPU | N | Valid | 1148 | 1148 | 1148 |
|  |  | Lost | 0 | 0 | 0 |
|  | Medium | | 76,00 | 16,94 | 10,67 |
|  | Standard deviation | | 14,571 | 1,647 | 9,598 |
|  | Minimum | | 19 | 15 | 1 |
|  | Maximum | | 102 | 20 | 140 |
|  | Percentiles | 25 | 69,25 | 16,00 | 5,00 |
|  |  | 50 | 80,00 | 17,00 | 9,00 |
|  |  | 75 | 86,00 | 18,00 | 13,00 |

## split file off.

FREQUENCIES VARIABLES= Nº_UPP_INTRAH DIA_1_UPP_INTRAHOSP_GROUPED

/ORDER=ANALYSIS.

**Frequencies**

Notes

| Output created | Facts | 01-MAY-2019 16:05:48 |
| --- | --- | --- |
| Comments |  |  |
| Entrance |  | C: Users. Diaz\Desktop\ELIA 1 5 2019\BBDD 1260.sav |
|  | Set of | Data_set2 |
|  | Filter | <none> |
|  | Weighting | <none> |
|  | Segment | <none> |
|  | N of rows in the  file of | 1260 |
| Management of lost value | Definition  absence | User-defined missing values are treated as lost. |
|  | Cases used | The statistics are based on all cases with valid data. |
| Syntax | Time | FREQUENCIES VARIABLES= Nº_UPP_INTRAH DIA_1_UPP_INTRAHOSP_GROUP ADO  /ORDER=ANALYSIS. |
| Resources |  | 00:00:00,02 |
|  | Time | 00:00:00,01 |

Statistics

|  | | Nº TOTAL OF HAPU | FIRST DAY THE DECLARATION OF  HAPU (grouped) |
| --- | --- | --- | --- |
| N | Valid | 112 | 112 |
|  | Lost | 1148 | 1148 |

# Frequency Table

Nº TOTAL OF HAPU

|  | | Frequency | Percentage | Valid percentage | Cumulative percentage |
| --- | --- | --- | --- | --- | --- |
| Valid | 1 | 97 | 7,7 | 86,6 | 86,6 |
|  | 2 | 13 | 1,0 | 11,6 | 98,2 |
|  | 3 | 2 | ,2 | 1,8 | 100,0 |
|  | Total | 112 | 8,9 | 100,0 |  |
| Lost | System | 1148 | 91,1 |  |  |
| Total | | 1260 | 100,0 |  |  |

FIRST DAY THE DECLARATION OF HAPU (grouped)

|  | | Frequency | Percentage | Valid percentage | Cumulative percentage |
| --- | --- | --- | --- | --- | --- |
| Valid | 1 -2 | 35 | 2,8 | 31,3 | 31,3 |
|  | 3 - 5 | 25 | 2,0 | 22,3 | 53,6 |
|  | 6 - 8 | 13 | 1,0 | 11,6 | 65,2 |
|  | 9 - 11 | 10 | ,8 | 8,9 | 74,1 |
|  | 12 - 14 | 7 | ,6 | 6,3 | 80,4 |
|  | 15 - 17 | 3 | ,2 | 2,7 | 83,0 |
|  | 18+ | 19 | 1,5 | 17,0 | 100,0 |
|  | Total | 112 | 8,9 | 100,0 |  |
| Lost | System | 1148 | 91,1 |  |  |
| Total | | 1260 | 100,0 |  |  |

## FREQUENCIES VARIABLES=DIA_1ºUPP_INTRAH

/FORMAT=NOTABLE

/NTILES=4

/STATISTICS=STDDEV MINIMUM MAXIMUM MEAN

/ORDER=ANALYSIS.

**Frequencies**

Notes

| Output created | Facts | 01-MAY-2019 16:05:56 |
| --- | --- | --- |
| Comments |  |  |
| Entrance |  | C: Users. Diaz\Desktop\ELIA 1 5 2019\BBDD 1260.sav |
|  | Set of | Data_set2 |
|  | Filter | <none> |
|  | Weighting | <none> |
|  | Segment | <none> |
|  | N of rows in the  file of | 1260 |
| Management of lost value | Definition  absence | User-defined missing values are treated as lost. |
|  | Cases used | The statistics are based on all cases with valid data. |
| Syntax | Time | FREQUENCIES VARIABLES=DIA_1ºUPP_INTRAH  /FORMAT=NOTABLE  /NTILES=4  /STATISTICS=STDDEV MINIMUM MAXIMUM MEAN  /ORDER=ANALYSIS. |
| Resources |  | 00:00:00,02 |
|  | Time | 00:00:00,02 |

Statistics

| N | Valid | 112 |
| --- | --- | --- |
|  | Lost | 1148 |
| Medium | | 11,74 |
| Standard deviation | | 13,605 |
| Minimum | | 0 |
| Maximum | | 61 |
| Percentiles | 25 | 3,00 |
|  | 50 | 6,00 |
|  | 75 | 14,00 |

## * Export results.

OUTPUT EXPORT

/CONTENTS EXPORT=ALL LAYERS=PRINTSETTING MODELVIEWS=PRINTSETTING

/XLS DOCUMENTFILE='C: Users\Im. Diaz\Desktop\ELIA 1 5 2019\CATEGORY AND LOCATION ELIA 1 5 '+ '2019.xls'

OPERATION=CREATEFILE SHEET='UPPI Norton >14'

|  | | n_HAPU | | | | | | | | | | | |
| --- | --- | --- | --- | --- | --- | --- | --- | --- | --- | --- | --- | --- | --- |
|  |  | 1 | | | 2 | | | 3 | | | Total | | |
|  |  | Counting | of column N | of table N | Counting | of column N | of table N | Counting | of column N | of table N | Counting | of N  column | of table N |
| CATEGORY_ 1ST HAPU | CATEGORY_1 | 58 | 51,8% | 45,0% | 11 | 73,3% | 8,5% | 2 | 100,0% | 1,6% | 71 | 55,0% | 55,0% |
|  | CATEGORY_2 | 51 | 45,5% | 39,5% | 4 | 26,7% | 3,1% | 0 | 0,0% | 0,0% | 55 | 42,6% | 42,6% |
|  | CATEGORY_3 | 3 | 2,7% | 2,3% | 0 | 0,0% | 0,0% | 0 | 0,0% | 0,0% | 3 | 2,3% | 2,3% |
|  | CATEGORY_4 | 0 | 0,0% | 0,0% | 0 | 0,0% | 0,0% | 0 | 0,0% | 0,0% | 0 | 0,0% | 0,0% |
|  | NO VALUE | 0 | 0,0% | 0,0% | 0 | 0,0% | 0,0% | 0 | 0,0% | 0,0% | 0 | 0,0% | 0,0% |
|  | Total | 112 | 100,0% | 86,8% | 15 | 100,0% | 11,6% | 2 | 100,0% | 1,6% | 129 | 100,0% | 100,0% |

|  | | | | n_HAPU | | | | | | | |
| --- | --- | --- | --- | --- | --- | --- | --- | --- | --- | --- | --- |
|  |  |  |  | 1 | | 2 | | 3 | | Total | |
|  |  |  |  | Counting | of table N | Counting | of N of  table | Counting | of N of  table | Counting | of table N |
| CATEGORY _1ª HAPU | CATEGORY_1 | LOCALIZATION_1ª HAPU | WITHOUT HAPU | 0 | 0,0% | 0 | 0,0% | 0 | 0,0% | 0 | 0,0% |
|  | | | occiput | 1 | 1,4% | 0 | 0,0% | 0 | 0,0% | 1 | 1,4% |
|  |  |  | shoulder blade | 1 | 1,4% | 0 | 0,0% | 0 | 0,0% | 1 | 1,4% |
|  |  |  | elbow | 2 | 2,8% | 1 | 1,4% | 0 | 0,0% | 3 | 4,2% |
|  | | | sacralcoxis | 27 | 38,0% | 1 | 1,4% | 1 | 1,4% | 29 | 40,8% |
|  |  |  | heel | 19 | 26,8% | 6 | 8,5% | 1 | 1,4% | 26 | 36,6% |
|  |  |  | toes | 0 | 0,0% | 0 | 0,0% | 0 | 0,0% | 0 | 0,0% |
|  |  |  | ear | 0 | 0,0% | 0 | 0,0% | 0 | 0,0% | 0 | 0,0% |
|  |  |  | shoulder | 0 | 0,0% | 0 | 0,0% | 0 | 0,0% | 0 | 0,0% |
|  |  |  | elbow | 0 | 0,0% | 0 | 0,0% | 0 | 0,0% | 0 | 0,0% |
|  |  |  | iliac crest | 0 | 0,0% | 0 | 0,0% | 0 | 0,0% | 0 | 0,0% |
|  | | | trochanter | 1 | 1,4% | 0 | 0,0% | 0 | 0,0% | 1 | 1,4% |
|  |  |  | knee (L.external) | 0 | 0,0% | 0 | 0,0% | 0 | 0,0% | 0 | 0,0% |
|  |  |  | knee (inner l.) | 0 | 0,0% | 0 | 0,0% | 0 | 0,0% | 0 | 0,0% |
|  | | | internal malleolus | 3 | 4,2% | 1 | 1,4% | 0 | 0,0% | 4 | 5,6% |
|  |  |  | exterminal malleolus | 1 | 1,4% | 1 | 1,4% | 0 | 0,0% | 2 | 2,8% |
|  |  |  | elbow (p. ventral) | 0 | 0,0% | 0 | 0,0% | 0 | 0,0% | 0 | 0,0% |
|  |  |  | rib | 0 | 0,0% | 0 | 0,0% | 0 | 0,0% | 0 | 0,0% |
|  |  |  | front face of the thigh | 0 | 0,0% | 0 | 0,0% | 0 | 0,0% | 0 | 0,0% |
|  |  |  | knee (front) | 0 | 0,0% | 0 | 0,0% | 0 | 0,0% | 0 | 0,0% |
|  |  |  | toes (front) | 0 | 0,0% | 0 | 0,0% | 0 | 0,0% | 0 | 0,0% |
|  |  |  | other | 3 | 4,2% | 1 | 1,4% | 0 | 0,0% | 4 | 5,6% |
|  |  |  | Total | 58 | 81,7% | 11 | 15,5% | 2 | 2,8% | 71 | 100,0% |

|  | | | | n_HAPU | | | | | |
| --- | --- | --- | --- | --- | --- | --- | --- | --- | --- |
|  |  |  |  | 1 | | 2 | | Total | |
|  |  |  |  | Counting | of table N | Counting | of N of  table | Counting | of N of  table |
| CATEGORY _1ª HAPU | CATEGORY_2 | LOCALIZATION_1ª HAPU | WITHOUT HAPU | 0 | 0,0% | 0 | 0,0% | 0 | 0,0% |
|  |  |  | occiput | 0 | 0,0% | 0 | 0,0% | 0 | 0,0% |
|  |  |  | shoulder blade | 0 | 0,0% | 0 | 0,0% | 0 | 0,0% |
|  | | | elbow | 2 | 3,6% | 0 | 0,0% | 2 | 3,6% |
|  |  |  | sacralcoxis | 28 | 50,9% | 2 | 3,6% | 30 | 54,5% |
|  |  |  | heel | 11 | 20,0% | 1 | 1,8% | 12 | 21,8% |
|  |  |  | toes | 1 | 1,8% | 0 | 0,0% | 1 | 1,8% |
|  |  |  | ear | 0 | 0,0% | 0 | 0,0% | 0 | 0,0% |
|  |  |  | shoulder | 0 | 0,0% | 0 | 0,0% | 0 | 0,0% |
|  |  |  | elbow | 0 | 0,0% | 0 | 0,0% | 0 | 0,0% |
|  |  |  | iliac crest | 0 | 0,0% | 0 | 0,0% | 0 | 0,0% |
|  | | | trochanter | 1 | 1,8% | 0 | 0,0% | 1 | 1,8% |
| knee (L.external) | | | | 0 | 0,0% | 0 | 0,0% | 0 | 0,0% |
|  | | | knee (inner l.) | 1 | 1,8% | 0 | 0,0% | 1 | 1,8% |
|  |  |  | internal malleolus | 2 | 3,6% | 0 | 0,0% | 2 | 3,6% |
|  |  |  | exterminal malleolus | 3 | 5,5% | 1 | 1,8% | 4 | 7,3% |
|  |  |  | elbow (p. ventral) | 0 | 0,0% | 0 | 0,0% | 0 | 0,0% |
|  |  |  | rib | 0 | 0,0% | 0 | 0,0% | 0 | 0,0% |
|  |  |  | front face of the thigh | 0 | 0,0% | 0 | 0,0% | 0 | 0,0% |
|  |  |  | knee (front) | 0 | 0,0% | 0 | 0,0% | 0 | 0,0% |
|  |  |  | toes (front) | 0 | 0,0% | 0 | 0,0% | 0 | 0,0% |
|  |  |  | other | 2 | 3,6% | 0 | 0,0% | 2 | 3,6% |
|  |  |  | Total | 51 | 92,7% | 4 | 7,3% | 55 | 100,0% |

|  | | | | n_HAPU | | | |  | | | | | |
| --- | --- | --- | --- | --- | --- | --- | --- | --- | --- | --- | --- | --- | --- |
|  |  |  |  | 1 | | Total | |  |  |  |  |  |  |
|  |  |  |  | Counting | of table N | Counting | of N of  table |  |  |  |  |  |  |
| CATEGORY_HAPU | CATEGORY_3 | LOCALIZATION OF THE 1ST HAPU | WITHOUT HAPU | 0 | 0,0% | 0 | 0,0% |  |  |  |  |  |  |
|  |  |  | occiput | 0 | 0,0% | 0 | 0,0% |  |  |  |  |  |  |
|  |  |  | shoulder blade | 0 | 0,0% | 0 | 0,0% |  |  |  |  |  |  |
|  |  |  | elbow | 0 | 0,0% | 0 | 0,0% |  |  |  |  |  |  |
|  |  |  | sacralcoxis | 2 | 66,7% | 2 | 66,7% |  |  |  |  |  |  |
|  |  |  | heel | 1 | 33,3% | 1 | 33,3% |  |  |  |  |  |  |
|  |  |  | toes | 0 | 0,0% | 0 | 0,0% |  |  |  |  |  |  |
|  |  |  | ear | 0 | 0,0% | 0 | 0,0% |  |  |  |  |  |  |
|  |  |  | shoulder | 0 | 0,0% | 0 | 0,0% |  |  |  |  |  |  |
|  |  |  | elbow | 0 | 0,0% | 0 | 0,0% |  |  |  |  |  |  |
|  |  |  | iliac crest | 0 | 0,0% | 0 | 0,0% |  |  |  |  |  |  |
|  |  |  | trochanter | 0 | 0,0% | 0 | 0,0% |  |  |  |  |  |  |
|  |  |  | knee (L.external) | 0 | 0,0% | 0 | 0,0% |  |  |  |  |  |  |
|  |  |  | knee (inner l.) | 0 | 0,0% | 0 | 0,0% |  |  |  |  |  |  |
|  |  |  | internal malleolus | 0 | 0,0% | 0 | 0,0% |  |  |  |  |  |  |
|  |  |  | exterminal malleolus | 0 | 0,0% | 0 | 0,0% |  |  |  |  |  |  |
|  |  |  | elbow (p. ventral) | 0 | 0,0% | 0 | 0,0% |  |  |  |  |  |  |
|  |  |  | rib | 0 | 0,0% | 0 | 0,0% |  |  |  |  |  |  |
|  |  |  | front face of the thigh | 0 | 0,0% | 0 | 0,0% |  |  |  |  |  |  |
|  |  |  | knee (front) | 0 | 0,0% | 0 | 0,0% |  |  |  |  |  |  |
|  |  |  | toes (front) | 0 | 0,0% | 0 | 0,0% |  |  |  |  |  |  |
|  |  |  | other | 0 | 0,0% | 0 | 0,0% |  |  |  |  |  |  |
|  |  |  | Total | 3 | 100,0% | 3 | 100,0% |  |  |  |  |  |  |
|  | | | | | | | | | | | | | |
|  | | DAYS OF HOSPITAL | | | | | | | |  | | | |
|  |  | Counting | Medium | Deviation  standard | Maximum | Minimum | Medium | 25th percentile | 75th percentile |  |  |  |  |
| PATIENTS IN HAPU PROTOCOL | WITH HAPU | 112 | 21 | 19 | 113 | 2 | 15 | 9 | 29 |  |  |  |  |
|  | WITHOUT  HAPU | 1148 | 11 | 10 | 140 | 1 | 9 | 5 | 13 |  |  |  |  |
|  | Total | 1260 | 12 | 11 | 140 | 1 | 9 | 6 | 14 |  |  |  |  |
|  | | | | | | | | | | | | | |
|  | | n_HAPU | | | | | | | | | | | |
|  |  | **1** | | | **2** | | | **3** | | | Total | | |
|  |  | Counting | of column N | of table N | Counting | of column N | of table N | Counting | of column N | of table N | Counting | of N  column | of table N |
| LOCALIZATION_HAPU | WITHOUT  HAPU | 0 | 0,0% | 0,0% | 0 | 0,0% | 0,0% | 0 | 0,0% | 0,0% | 0 | 0,0% | 0,0% |
|  | occiput | 1 | ,9% | ,8% | 0 | 0,0% | 0,0% | 0 | 0,0% | 0,0% | 1 | ,8% | ,8% |
|  | shoulder blade | 1 | ,9% | ,8% | 0 | 0,0% | 0,0% | 0 | 0,0% | 0,0% | 1 | ,8% | ,8% |
|  | elbow | 4 | 3,6% | 3,1% | 1 | 6,7% | ,8% | 0 | 0,0% | 0,0% | 5 | 3,9% | 3,9% |
|  | sacralcoxis | 57 | 50,9% | 44,2% | 3 | 20,0% | 2,3% | 1 | 50,0% | ,8% | 61 | 47,3% | 47,3% |
|  | heel | 31 | 27,7% | 24,0% | 7 | 46,7% | 5,4% | 1 | 50,0% | ,8% | 39 | 30,2% | 30,2% |
|  | toes | 1 | ,9% | ,8% | 0 | 0,0% | 0,0% | 0 | 0,0% | 0,0% | 1 | ,8% | ,8% |
|  | ear | 0 | 0,0% | 0,0% | 0 | 0,0% | 0,0% | 0 | 0,0% | 0,0% | 0 | 0,0% | 0,0% |
|  | shoulder | 0 | 0,0% | 0,0% | 0 | 0,0% | 0,0% | 0 | 0,0% | 0,0% | 0 | 0,0% | 0,0% |
|  | elbow | 0 | 0,0% | 0,0% | 0 | 0,0% | 0,0% | 0 | 0,0% | 0,0% | 0 | 0,0% | 0,0% |
|  | iliac crest | 0 | 0,0% | 0,0% | 0 | 0,0% | 0,0% | 0 | 0,0% | 0,0% | 0 | 0,0% | 0,0% |
|  | trochanter | 2 | 1,8% | 1,6% | 0 | 0,0% | 0,0% | 0 | 0,0% | 0,0% | 2 | 1,6% | 1,6% |
| knee (L.external) | | 0 | 0,0% | 0,0% | 0 | 0,0% | 0,0% | 0 | 0,0% | 0,0% | 0 | 0,0% | 0,0% |
|  | knee (inner l.) | 1 | ,9% | ,8% | 0 | 0,0% | 0,0% | 0 | 0,0% | 0,0% | 1 | ,8% | ,8% |
|  | internal malleolus | 5 | 4,5% | 3,9% | 1 | 6,7% | ,8% | 0 | 0,0% | 0,0% | 6 | 4,7% | 4,7% |
|  | exterminal malleolus | 4 | 3,6% | 3,1% | 2 | 13,3% | 1,6% | 0 | 0,0% | 0,0% | 6 | 4,7% | 4,7% |
|  | elbow (p. ventral) | 0 | 0,0% | 0,0% | 0 | 0,0% | 0,0% | 0 | 0,0% | 0,0% | 0 | 0,0% | 0,0% |
|  | rib | 0 | 0,0% | 0,0% | 0 | 0,0% | 0,0% | 0 | 0,0% | 0,0% | 0 | 0,0% | 0,0% |
|  | front face of the thigh | 0 | 0,0% | 0,0% | 0 | 0,0% | 0,0% | 0 | 0,0% | 0,0% | 0 | 0,0% | 0,0% |
|  | knee (front) | 0 | 0,0% | 0,0% | 0 | 0,0% | 0,0% | 0 | 0,0% | 0,0% | 0 | 0,0% | 0,0% |
|  | toes (front) | 0 | 0,0% | 0,0% | 0 | 0,0% | 0,0% | 0 | 0,0% | 0,0% | 0 | 0,0% | 0,0% |
|  | other | 5 | 4,5% | 3,9% | 1 | 6,7% | ,8% | 0 | 0,0% | 0,0% | 6 | 4,7% | 4,7% |
| Total | | 112 | 100,0% | 86,8% | 15 | 100,0% | 11,6% | 2 | 100,0% | 1,6% | 129 | 100,0% | 100,0% |
|  | | | | | | | | | | | | | |
|  | | PATIENTS IN HAPU PROTOCOL | | | | | | | | |  | | |
|  |  | WITH HAPU | | | WITHOUT HAPU | | | Total | | |  |  |  |
|  |  | Counting | of column N | of table N | Counting | of column N | of table N | Counting | of column N | of table N |  |  |  |
| AGE_GRUP_3 | < 65 YEARS | 19 | 17,0% | 1,5% | 215 | 18,7% | 17,1% | 234 | 18,6% | 18,6% |  |  |  |
|  | 66 to 79 YEARS | 58 | 51,8% | 4,6% | 613 | 53,4% | 48,7% | 671 | 53,3% | 53,3% |  |  |  |
|  | > 80 YEARS | 35 | 31,3% | 2,8% | 320 | 27,9% | 25,4% | 355 | 28,2% | 28,2% |  |  |  |
|  | no data | 0 | 0,0% | 0,0% | 0 | 0,0% | 0,0% | 0 | 0,0% | 0,0% |  |  |  |
|  | Total | 112 | 100,0% | 8,9% | 1148 | 100,0% | 91,1% | 1260 | 100,0% | 100,0% |  |  |  |
|  | | | | | | | | | | | | | |
|  | | PATIENTS IN HAPU PROTOCOL | | | | | | | | |  | | |
|  |  | WITH HAPU | | | WITHOUT HAPU | | | Total | | |  |  |  |
|  |  | Counting | of column N | of table N | Counting | of column N | of table N | Counting | of column N | of table N |  |  |  |
| DAYS_TOTAL_HOSPITA  LIZATION_GROUP_5 | < 7 DAYS | 12 | 10,7% | 1,0% | 405 | 35,3% | 32,1% | 417 | 33,1% | 33,1% |  |  |  |
|  | 7 to 14 DAYS | 41 | 36,6% | 3,3% | 497 | 43,3% | 39,4% | 538 | 42,7% | 42,7% |  |  |  |
|  | 15 to 21 DAYS | 22 | 19,6% | 1,7% | 160 | 13,9% | 12,7% | 182 | 14,4% | 14,4% |  |  |  |
|  | 22 to 28 DAYS | 9 | 8,0% | ,7% | 51 | 4,4% | 4,0% | 60 | 4,8% | 4,8% |  |  |  |
|  | > 28 DAYS | 28 | 25,0% | 2,2% | 35 | 3,0% | 2,8% | 63 | 5,0% | 5,0% |  |  |  |
|  | NO DATA | 0 | 0,0% | 0,0% | 0 | 0,0% | 0,0% | 0 | 0,0% | 0,0% |  |  |  |
|  | Total | 112 | 100,0% | 8,9% | 1148 | 100,0% | 91,1% | 1260 | 100,0% | 100,0% |  |  |  |
|  | | | | | | | | | | | | | |
|  | | PATIENTS IN HAPU PROTOCOL | | | | | | | | |  | | |
|  |  | WITH HAPU | | | WITHOUT HAPU | | | Total | | |  |  |  |
|  |  | Counting | of column N | of table N | Counting | of column N | of table N | Counting | of column N | of table N |  |  |  |
| RISK_FACTORS_RR_7 | 7A 10 RF | 6 | 5,4% | ,5% | 133 | 11,6% | 10,6% | 139 | 11,0% | 11,0% |  |  |  |
|  | 0 to 6 RF | 106 | 94,6% | 8,4% | 1015 | 88,4% | 80,6% | 1121 | 89,0% | 89,0% |  |  |  |
|  | NO DATA | 0 | 0,0% | 0,0% | 0 | 0,0% | 0,0% | 0 | 0,0% | 0,0% |  |  |  |
|  | Total | 112 | 100,0% | 8,9% | 1148 | 100,0% | 91,1% | 1260 | 100,0% | 100,0% |  |  |  |
