# Supplementary material for: Incidence of hospital-acquired pressure ulcers in patients with "minimal risk" according to the "Norton-MI" scale
Source: PLoS One. 2020 Jan 8;15(1):e0227052. doi: 10.1371/journal.pone.0227052 (PMC6948734; doi:10.1371/journal.pone.0227052)
Supplement: S2 File — (DOCX) [file pone.0227052.s002.docx]

**Logistical Regression**

| **Summary of case processing** | | | |
| --- | --- | --- | --- |
| Unweighted cases | | N | Percentage |
| Selected Cases | Included in the analysis | 1260 | 100,0 |
|  | Lost Cases | 0 | ,0 |
|  | Total | 1260 | 100,0 |
| Cases not selected | | 0 | ,0 |
| Total | | 1260 | 100,0 |

| a. If weighting is enabled, refer to the classification table for the total number of cases. |
| --- |

| **Coding of the dependent variable** | |
| --- | --- |
| Original value | Internal value |
| ,00 | 0 |
| 1,00 | 1 |

| **Coding of categorical variables** | | | |
| --- | --- | --- | --- |
|  | | Frequency | Parameter coding |
|  |  |  | (1) |
| GENDER | male | 598 | 1,000 |
|  | famele | 662 | ,000 |

**Block 0: Initial block**

| **Classification table^,b^** | | | | | |
| --- | --- | --- | --- | --- | --- |
|  | Observed | | Forecast | | |
|  |  |  | HAPU | | Correct Percentage |
|  |  |  | ,00 | 1,00 |  |
| Step 0 | HAPU | ,00 | 1148 | 0 | 100,0 |
|  |  | 1,00 | 112 | 0 | ,0 |
|  | Overall percentage | |  |  | 91,1 |

| a. A constant is included in the model. |
| --- |
| b. The cut-off value is ,500 |

| **Variables in the equation** | | | | | | | |
| --- | --- | --- | --- | --- | --- | --- | --- |
|  | | B | E.T. | Wald | g | Next | Exp(B) |
| Step 0 | Constant | -2,327 | ,099 | 552,695 | 1 | ,000 | ,098 |

| **Variables not in the equation** | | | | | |
| --- | --- | --- | --- | --- | --- |
|  | | | Score | g | Next |
| Step 0 | Variables | GENDER(1) | 1,841 | 1 | ,175 |
|  | Global statistics | | 1,841 | 1 | ,175 |

**Block 1: Method = Enter**

| **Omnibus tests on model coefficients** | | | | |
| --- | --- | --- | --- | --- |
|  | | Chi square | g | Next |
| Step 1 | Step | 1,838 | 1 | ,175 |
|  | Block | 1,838 | 1 | ,175 |
|  | Model | 1,838 | 1 | ,175 |

| **Model Overview** | | | |
| --- | --- | --- | --- |
| Step | -2 plausibility log | R square of Cox and Snell | Nagelkerke Square R |
| 1 | 754,060a | ,001 | ,003 |

| a. The estimate has ended in iteration number 5 because the parameter estimates have changed by less than ,001. |
| --- |

| **Classification table** | | | | | |
| --- | --- | --- | --- | --- | --- |
|  | Observed | | Forecast | | |
|  |  |  | HAPU | | Correct Percentage |
|  |  |  | ,00 | 1,00 |  |
| Step 1 | HAPU | ,00 | 1148 | 0 | 100,0 |
|  |  | 1,00 | 112 | 0 | ,0 |
|  | Overall percentage | |  |  | 91,1 |

| a. The cut-off value is ,500 |
| --- |

| **Variables in the equation** | | | | | | | |
| --- | --- | --- | --- | --- | --- | --- | --- |
|  | | B | E.T. | Wald | g | Next | Exp(B) |
|  |  |  |  |  |  |  |  |
| Step 1a | GENDER(1) | ,269 | ,198 | 1,833 | 1 | ,176 | 1,308 |
|  | Constant | -2,462 | ,144 | 290,487 | 1 | ,000 | ,085 |

| **Variables in the equation** | | | |
| --- | --- | --- | --- |
|  | | C.I. 95% for EXP(B) | |
|  |  | Lower | Superior |
| Step 1a | GENDER(1) | ,887 | 1,930 |
|  | Constant |  |  |

| a. Variable(s) entered in step 1: GENDER. |
| --- |

**Logistical Regression**

| **Summary of case processing** | | | |
| --- | --- | --- | --- |
| Unweighted cases | | N | Percentage |
| Selected Cases | Included in the analysis | 1260 | 100,0 |
|  | Lost Cases | 0 | ,0 |
|  | Total | 1260 | 100,0 |
| Cases not selected | | 0 | ,0 |
| Total | | 1260 | 100,0 |

| 1. If weighting is enabled, refer to the classification table for the total number of cases. |
| --- |

| **Coding of the dependent variable** | |
| --- | --- |
| Original value | Internal value |
| ,00 | 0 |
| 1,00 | 1 |

| **Coding of categorical variables** | | | |
| --- | --- | --- | --- |
|  | | Frequency | Parameter coding |
|  |  |  | (1) |
| FR_6_10 | 0-5 | 826 | ,000 |
|  | 6 or more | 434 | 1,000 |

**Block 0: Initial block**

| **Classification table^,b^** | | | | | |
| --- | --- | --- | --- | --- | --- |
|  | Observed | | Forecast | | |
|  |  |  | HAPU | | Correct Percentage |
|  |  |  | ,00 | 1,00 |  |
| Step 0 | HAPU | ,00 | 1148 | 0 | 100,0 |
|  |  | 1,00 | 112 | 0 | ,0 |
|  | Overall percentage | |  |  | 91,1 |

| 1. A constant is included in the model. |
| --- |
| 1. The cut-off value is ,500 |

| **Variables in the equation** | | | | | | | |
| --- | --- | --- | --- | --- | --- | --- | --- |
|  | | B | E.T. | Wald | g | Next | Exp(B) |
| Step 0 | Constant | -2,327 | ,099 | 552,695 | 1 | ,000 | ,098 |

| **Variables not in the equation** | | | | | |
| --- | --- | --- | --- | --- | --- |
|  | | | Score | g | Next |
| Step 0 | Variables | FR_6_10(1) | ,014 | 1 | ,904 |
|  | Global statistics | | ,014 | 1 | ,904 |

**Block 1: Method = Enter**

| **Omnibus tests on model coefficients** | | | | |
| --- | --- | --- | --- | --- |
|  | | Chi square | g | Next |
| Step 1 | Step | ,015 | 1 | ,904 |
|  | Block | ,015 | 1 | ,904 |
|  | Model | ,015 | 1 | ,904 |

| **Model Overview** | | | |
| --- | --- | --- | --- |
| Step | -2 plausibility log | R square of Cox and Snell | Nagelkerke Square R |
| 1 | 755,884a | ,000 | ,000 |

| a. The estimate has ended in iteration number 5 because the parameter estimates have changed by less than ,001. |
| --- |

| **Classification table** | | | | | |
| --- | --- | --- | --- | --- | --- |
|  | Observed | | Forecast | | |
|  |  |  | HAPU | | Correct Percentage |
|  |  |  | ,00 | 1,00 |  |
| Step 1 | HAPU | ,00 | 1148 | 0 | 100,0 |
|  |  | 1,00 | 112 | 0 | ,0 |
|  | Overall percentage | |  |  | 91,1 |

| a. The cut-off value is ,500 |
| --- |

| **Variables in the equation** | | | | | | | |
| --- | --- | --- | --- | --- | --- | --- | --- |
|  | | B | E.T. | Wald | g | Next | Exp(B) |
|  |  |  |  |  |  |  |  |
| Step 1a | FR_6_10(1) | -,025 | ,209 | ,014 | 1 | ,904 | ,975 |
|  | Constant | -2,319 | ,122 | 362,200 | 1 | ,000 | ,098 |

| **Variables in the equation** | | | |
| --- | --- | --- | --- |
|  | | C. I. 95% for EXP(B) | |
|  |  | Lower | Superior |
| Step 1a | FR_6_10(1) | ,647 | 1,469 |
|  | Constant |  |  |

| 1. Variable(s) entered in step 1: FR_6_10. |
| --- |

**Logistical Regression**

| **Summary of case processing** | | | |
| --- | --- | --- | --- |
| Unweighted cases | | N | Percentage |
| Selected Cases | Included in the analysis | 1260 | 100,0 |
|  | Lost Cases | 0 | ,0 |
|  | Total | 1260 | 100,0 |
| Cases not selected | | 0 | ,0 |
| Total | | 1260 | 100,0 |

| 1. If weighting is enabled, refer to the classification table for the total number of cases. | | |
| --- | --- | --- |
| **Coding of the dependent variable** | |  |
| Original value | Internal value |  |
| ,00 | 0 |  |
| 1,00 | 1 |  |

| **Coding of categorical variables** | | | |
| --- | --- | --- | --- |
|  | | Frequency | Parameter coding |
|  |  |  | (1) |
| ALT. MOBILITY | NO | 361 | ,000 |
|  | YES | 899 | 1,000 |

**Block 0: Initial block**

| **Classification table^,b^** | | | | | |
| --- | --- | --- | --- | --- | --- |
|  | Observed | | Forecast | | |
|  |  |  | HAPU | | Correct Percentage |
|  |  |  | ,00 | 1,00 |  |
| Step 0 | HAPU | ,00 | 1148 | 0 | 100,0 |
|  |  | 1,00 | 112 | 0 | ,0 |
|  | Overall percentage | |  |  | 91,1 |

| a. A constant is included in the model. | | | | | | |  |  |
| --- | --- | --- | --- | --- | --- | --- | --- | --- |
| b. The cut-off value is ,500 | | | | | | |  |  |
| **Variables in the equation** | | | | | | | | |
|  | | B | E.T. | Wald | g | Next | | Exp(B) |
| Step 0 | Constant | -2,327 | ,099 | 552,695 | 1 | ,000 | | ,098 |

| **Variables not in the equation** | | | | | |
| --- | --- | --- | --- | --- | --- |
|  | | | Score | g | Next |
| Step 0 | Variables | ALT_MOBILITY(1) | 3,000 | 1 | ,083 |
|  | Global statistics | | 3,000 | 1 | ,083 |

**Block 1: Method = Enter**

| **Omnibus tests on model coefficients** | | | | |
| --- | --- | --- | --- | --- |
|  | | Chi square | g | Next |
| Step 1 | Step | 2,886 | 1 | ,089 |
|  | Block | 2,886 | 1 | ,089 |
|  | Model | 2,886 | 1 | ,089 |

| **Model Overview** | | | |
| --- | --- | --- | --- |
| Step | -2 plausibility log | R square of Cox and Snell | Nagelkerke Square R |
| 1 | 753,012a | ,002 | ,005 |

| a. The estimate has ended in iteration number 5 because the parameter estimates have changed by less than ,001. |
| --- |

| **Classification table** | | | | | |
| --- | --- | --- | --- | --- | --- |
|  | Observed | | Forecast | | |
|  |  |  | HAPU | | Correct Percentage |
|  |  |  | ,00 | 1,00 |  |
| Step 1 | HAPU | ,00 | 1148 | 0 | 100,0 |
|  |  | 1,00 | 112 | 0 | ,0 |
|  | Overall percentage | |  |  | 91,1 |

| 1. The cut-off value is ,500 |
| --- |

| **Variables in the equation** | | | | | | |
| --- | --- | --- | --- | --- | --- | --- |
|  | | B | E.T. | Wald | g | Next |
|  |  |  |  |  |  |  |
| Step 1a | ALT_MOBILITY(1) | -,359 | ,208 | 2,975 | 1 | ,085 |
|  | Constant | -2,083 | ,168 | 154,260 | 1 | ,000 |

| **Variables in the equation** | | | | |
| --- | --- | --- | --- | --- |
|  | | Exp(B) | C. I. 95% for EXP(B) | |
|  |  |  | Lower | Superior |
| Step 1a | ALT_DE_LA_MOBILITY(1) | ,699 | ,465 | 1,050 |
|  | Constant | ,125 |  |  |

| a. Variable(s) entered in step 1: ALT_MOBILITY. |
| --- |

**Logistical Regression**

| **Summary of case processing** | | | |
| --- | --- | --- | --- |
| Unweighted cases | | N | Percentage |
| Selected Cases | Included in the analysis | 1260 | 100,0 |
|  | Lost Cases | 0 | ,0 |
|  | Total | 1260 | 100,0 |
| Cases not selected | | 0 | ,0 |
| Total | | 1260 | 100,0 |

| a. If weighting is enabled, refer to the classification table for the total number of cases. |
| --- |

| **Coding of the dependent variable** | |
| --- | --- |
| Original value | Internal value |
| ,00 | 0 |
| 1,00 | 1 |

| **Coding of categorical variables** | | | |
| --- | --- | --- | --- |
|  | | Frequency | Parameter coding |
|  |  |  | (1) |
| ALT_ACTIVITY | NO | 408 | ,000 |
|  | YES | 852 | 1,000 |

**Block 0: Initial block**

| **Classification table^,b^** | | | | | |
| --- | --- | --- | --- | --- | --- |
|  | Observed | | Forecast | | |
|  |  |  | HAPU | | Correct Percentage |
|  |  |  | ,00 | 1,00 |  |
| Step 0 | HAPU | ,00 | 1148 | 0 | 100,0 |
|  |  | 1,00 | 112 | 0 | ,0 |
|  | Overall percentage | |  |  | 91,1 |

| a. A constant is included in the model. |
| --- |
| b. The cut-off value is ,500 |

| **Variables in the equation** | | | | | | | |
| --- | --- | --- | --- | --- | --- | --- | --- |
|  | | B | E.T. | Wald | g | Next | Exp(B) |
| Step 0 | Constant | -2,327 | ,099 | 552,695 | 1 | ,000 | ,098 |

| **Variables not in the equation** | | | | | |
| --- | --- | --- | --- | --- | --- |
|  | | | Score | g | Next |
| Step 0 | Variables | ALT_ACTIVITY(1) | 1,758 | 1 | ,185 |
|  | Global statistics | | 1,758 | 1 | ,185 |

**Block 1: Method = Enter**

| **Omnibus tests on model coefficients** | | | | |
| --- | --- | --- | --- | --- |
|  | | Chi square | g | Next |
| Step 1 | Step | 1,812 | 1 | ,178 |
|  | Block | 1,812 | 1 | ,178 |
|  | Model | 1,812 | 1 | ,178 |

| **Model Overview** | | | |
| --- | --- | --- | --- |
| Step | -2 plausibility log | R square of Cox and Snell | Nagelkerke Square R |
| 1 | 754,086a | ,001 | ,003 |

| a. The estimate has ended in iteration number 5 because the parameter estimates have changed by less than ,001. |
| --- |

| **Classification table** | | | | | |
| --- | --- | --- | --- | --- | --- |
|  | Observed | | Forecast | | |
|  |  |  | HAPU | | Correct Percentage |
|  |  |  | ,00 | 1,00 |  |
| Step 1 | HAPU | ,00 | 1148 | 0 | 100,0 |
|  |  | 1,00 | 112 | 0 | ,0 |
|  | Overall percentage | |  |  | 91,1 |

| a. The cut-off value is ,500 |
| --- |

| **Variables in the equation** | | | | | | | |
| --- | --- | --- | --- | --- | --- | --- | --- |
|  | | B | E.T. | Wald | g | Next | Exp(B) |
|  |  |  |  |  |  |  |  |
| Step 1a | ALT_ACTIVITY(1) | ,294 | ,222 | 1,747 | 1 | ,186 | 1,342 |
|  | Constant | -2,534 | ,190 | 178,428 | 1 | ,000 | ,079 |

| **Variables in the equation** | | | |
| --- | --- | --- | --- |
|  | | C. I. 95% for EXP(B) | |
|  |  | Lower | Superior |
| Step 1a | ALT_ACTIVITY(1) | ,868 | 2,075 |
|  | Constant |  |  |

| a. Variable(s) entered in step 1: ALT_ACTIVITY. |
| --- |

**Logistical Regression**

| **Summary of case processing** | | | |
| --- | --- | --- | --- |
| Unweighted cases | | N | Percentage |
| Selected Cases | Included in the analysis | 1260 | 100,0 |
|  | Lost Cases | 0 | ,0 |
|  | Total | 1260 | 100,0 |
| Cases not selected | | 0 | ,0 |
| Total | | 1260 | 100,0 |

| a. If weighting is enabled, refer to the classification table for the total number of cases. |
| --- |

| **Variable coding**  **clerk** | |
| --- | --- |
| Original value | Internal value |
| ,00 | 0 |
| 1,00 | 1 |

| **Coding of categorical variables** | | | |
| --- | --- | --- | --- |
|  | | Frequency | Parameter coding |
|  |  |  | (1) |
| ALT_NUTRITION | NO | 532 | ,000 |
|  | YES | 728 | 1,000 |

**Block 0: Initial block**

| **Classification table^,b^** | | | | | |
| --- | --- | --- | --- | --- | --- |
|  | Observed | | Forecast | | |
|  |  |  | HAPU | | Correct Percentage |
|  |  |  | ,00 | 1,00 |  |
| Step 0 | HAPU | ,00 | 1148 | 0 | 100,0 |
|  |  | 1,00 | 112 | 0 | ,0 |
|  | Overall percentage | |  |  | 91,1 |

| a. A constant is included in the model. |
| --- |
| b. The cut-off value is ,500 |

| **Variables in the equation** | | | | | | | |
| --- | --- | --- | --- | --- | --- | --- | --- |
|  | | B | E.T. | Wald | g | Next | Exp(B) |
| Step 0 | Constant | -2,327 | ,099 | 552,695 | 1 | ,000 | ,098 |

| **Variables not in the equation** | | | | | |
| --- | --- | --- | --- | --- | --- |
|  | | | Score | g | Next |
| Step 0 | Variables | ALT_NUTRITION(1) | 40,395 | 1 | ,000 |
|  | Global statistics | | 40,395 | 1 | ,000 |

**Block 1: Method = Enter**

| **Omnibus tests on model coefficients** | | | | |
| --- | --- | --- | --- | --- |
|  | | Chi square | g | Next |
| Step 1 | Step | 40,249 | 1 | ,000 |
|  | Block | 40,249 | 1 | ,000 |
|  | Model | 40,249 | 1 | ,000 |

| **Model Overview** | | | |
| --- | --- | --- | --- |
| Step | -2 plausibility log | R square of Cox and Snell | Nagelkerke Square R |
| 1 | 715,649a | ,031 | ,070 |

| a. The estimate has ended in iteration number 6 because the parameter estimates have changed by less than ,001. |
| --- |

| **Classification table** | | | | | |
| --- | --- | --- | --- | --- | --- |
|  | Observed | | Forecast | | |
|  |  |  | HAPU | | Correct Percentage |
|  |  |  | ,00 | 1,00 |  |
| Step 1 | HAPU | ,00 | 1148 | 0 | 100,0 |
|  |  | 1,00 | 112 | 0 | ,0 |
|  | Overall percentage | |  |  | 91,1 |

| 1. The cut-off value is ,500 |
| --- |

| **Variables in the equation** | | | | | | | |
| --- | --- | --- | --- | --- | --- | --- | --- |
|  | | B | E.T. | Wald | g | Next | Exp(B) |
|  |  |  |  |  |  |  |  |
| Step 1a | ALT_NUTRITION(1) | -1,301 | ,216 | 36,314 | 1 | ,000 | ,272 |
|  | Constant | -1,746 | ,122 | 205,174 | 1 | ,000 | ,174 |

| **Variables in the equation** | | | |
| --- | --- | --- | --- |
|  | | C.I. 95% for EXP(B) | |
|  |  | Lower | Superior |
| Step 1a | ALT_NUTRITION(1) | ,178 | ,416 |
|  | Constant |  |  |

| a. Variable(s) entered in step 1: ALT_NUTRITION. |
| --- |

**Logistical Regression**

| **Summary of case processing** | | | |
| --- | --- | --- | --- |
| Unweighted cases | | N | Percentage |
| Selected Cases | Included in the analysis | 1260 | 100,0 |
|  | Lost Cases | 0 | ,0 |
|  | Total | 1260 | 100,0 |
| Cases not selected | | 0 | ,0 |
| Total | | 1260 | 100,0 |

| a. If weighting is enabled, refer to the classification table for the total number of cases. |
| --- |

| **Coding of the dependent variable** | |
| --- | --- |
| Original value | Internal value |
| ,00 | 0 |
| 1,00 | 1 |

| **Coding of categorical variables** | | | |
| --- | --- | --- | --- |
|  | | Frequency | Parameter coding |
|  |  |  | (1) |
| EXCESS PRESSURE | NO | 831 | ,000 |
|  | YES | 429 | 1,000 |

**Block 0: Initial block**

| **Classification table^,b^** | | | | | |
| --- | --- | --- | --- | --- | --- |
|  | Observed | | Forecast | | |
|  |  |  | HAPU | | Correct Percentage |
|  |  |  | ,00 | 1,00 |  |
| Step 0 | HAPU | ,00 | 1148 | 0 | 100,0 |
|  |  | 1,00 | 112 | 0 | ,0 |
|  | Overall percentage | |  |  | 91,1 |

| a. A constant is included in the model. |
| --- |
| b. The cut-off value is ,500 |

| **Variables in the equation** | | | | | | | |
| --- | --- | --- | --- | --- | --- | --- | --- |
|  | | B | E.T. | Wald | g | Next | Exp(B) |
| Step 0 | Constant | -2,327 | ,099 | 552,695 | 1 | ,000 | ,098 |

| **Variables not in the equation** | | | | | |
| --- | --- | --- | --- | --- | --- |
|  | | | Score | g | Next |
| Step 0 | Variables | EXCESS_PRESSURE(1) | 44,317 | 1 | ,000 |
|  | Global statistics | | 44,317 | 1 | ,000 |

**Block 1: Method = Enter**

| **Omnibus tests on model coefficients** | | | | |
| --- | --- | --- | --- | --- |
|  | | Chi square | g | Next |
| Step 1 | Step | 41,606 | 1 | ,000 |
|  | Block | 41,606 | 1 | ,000 |
|  | Model | 41,606 | 1 | ,000 |

| **Model Overview** | | | |
| --- | --- | --- | --- |
| Step | -2 plausibility log | R square of Cox and Snell | Nagelkerke Square R |
| 1 | 714,292a | ,032 | ,072 |

| a. The estimate has ended in iteration number 6 because the parameter estimates have changed by less than ,001. |
| --- |

| **Classification table** | | | | | |
| --- | --- | --- | --- | --- | --- |
|  | Observed | | Forecast | | |
|  |  |  | HAPU | | Correct Percentage |
|  |  |  | ,00 | 1,00 |  |
| Step 1 | HAPU | ,00 | 1148 | 0 | 100,0 |
|  |  | 1,00 | 112 | 0 | ,0 |
|  | Overall percentage | |  |  | 91,1 |

| a. The cut-off value is ,500 |
| --- |

| **Variables in the equation** | | | | | | |
| --- | --- | --- | --- | --- | --- | --- |
|  | | B | E.T. | Wald | g | Next |
|  |  |  |  |  |  |  |
| Step 1a | EXCESS_PRESSURE(1) | 1,298 | ,205 | 39,990 | 1 | ,000 |
|  | Constant | -2,933 | ,158 | 343,066 | 1 | ,000 |

| **Variables in the equation** | | | | |
| --- | --- | --- | --- | --- |
|  | | Exp(B) | C. I. 95% for EXP(B) | |
|  |  |  | Lower | Superior |
| Step 1a | EXCESS_PRESSURE(1) | 3,663 | 2,450 | 5,478 |
|  | Constant | ,053 |  |  |

| a. Variable(s) entered in step 1: EXCESS_PRESSURE. |
| --- |

**Logistical Regression**

| **Summary of case processing** | | | |
| --- | --- | --- | --- |
| Unweighted cases | | N | Percentage |
| Selected Cases | Included in the analysis | 1260 | 100,0 |
|  | Lost Cases | 0 | ,0 |
|  | Total | 1260 | 100,0 |
| Cases not selected | | 0 | ,0 |
| Total | | 1260 | 100,0 |

| a. If weighting is enabled, refer to the classification table for the total number of cases. |
| --- |

| **Coding of the dependent variable** | |
| --- | --- |
| Original value | Internal value |
| ,00 | 0 |
| 1,00 | 1 |

| **Coding of categorical variables** | | | |
| --- | --- | --- | --- |
|  | | Frequency | Parameter coding |
|  |  |  | (1) |
| AGE ≥ 65 YEARS | NO | 219 | ,000 |
|  | YES | 1041 | 1,000 |

**Block 0: Initial block**

| **Classification table^,b^** | | | | | |
| --- | --- | --- | --- | --- | --- |
|  | Observed | | Forecast | | |
|  |  |  | HAPU | | Correct Percentage |
|  |  |  | ,00 | 1,00 |  |
| Step 0 | HAPU | ,00 | 1148 | 0 | 100,0 |
|  |  | 1,00 | 112 | 0 | ,0 |
|  | Overall percentage | |  |  | 91,1 |

| a. A constant is included in the model. |
| --- |
| b. The cut-off value is ,500 |

| **Variables in the equation** | | | | | | | |
| --- | --- | --- | --- | --- | --- | --- | --- |
|  | | B | E.T. | Wald | g | Next | Exp(B) |
| Step 0 | Constant | -2,327 | ,099 | 552,695 | 1 | ,000 | ,098 |

| **Variables not in the equation** | | | | | |
| --- | --- | --- | --- | --- | --- |
|  | | | Score | g | Next |
| Step 0 | Variables | AGE ≥ 65 YEARS (1) | 1,362 | 1 | ,243 |
|  | Global statistics | | 1,362 | 1 | ,243 |

**Block 1: Method = Enter**

| **Omnibus tests on model coefficients** | | | | |
| --- | --- | --- | --- | --- |
|  | | Chi square | g | Next |
| Step 1 | Step | 1,446 | 1 | ,229 |
|  | Block | 1,446 | 1 | ,229 |
|  | Model | 1,446 | 1 | ,229 |

| **Model Overview** | | | |
| --- | --- | --- | --- |
| Step | -2 plausibility log | R square of Cox and Snell | Nagelkerke Square R |
| 1 | 754,452a | ,001 | ,003 |

| a. The estimate has ended in iteration number 5 because the parameter estimates have changed by less than ,001. |
| --- |

| **Classification table** | | | | | |
| --- | --- | --- | --- | --- | --- |
|  | Observed | | Forecast | | |
|  |  |  | HAPU | | Correct Percentage |
|  |  |  | ,00 | 1,00 |  |
| Step 1 | HAPU | ,00 | 1148 | 0 | 100,0 |
|  |  | 1,00 | 112 | 0 | ,0 |
|  | Overall percentage | |  |  | 91,1 |

| 1. The cut-off value is ,500 |
| --- |

| **Variables in the equation** | | | | | | | |
| --- | --- | --- | --- | --- | --- | --- | --- |
|  | | B | E.T. | Wald | g | Next | Exp(B) |
|  |  |  |  |  |  |  |  |
| Step 1a | AGE ≥ 65 YEARS (1) | ,335 | ,288 | 1,350 | 1 | ,245 | 1,397 |
|  | Constant | -2,610 | ,268 | 95,188 | 1 | ,000 | ,074 |

| **Variables in the equation** | | | |
| --- | --- | --- | --- |
|  | | C. I. 95% for EXP(B) | |
|  |  | Lower | Superior |
| Step 1a | AGE ≥ 65 YEARS (1) | ,795 | 2,457 |
|  | Constant |  |  |

| a. Variable(s) entered in step 1: AGE ≥ 65 YEARS . |
| --- |

**Logistical Regression**

| **Summary of case processing** | | | |
| --- | --- | --- | --- |
| Unweighted cases | | N | Percentage |
| Selected Cases | Included in the analysis | 1260 | 100,0 |
|  | Lost Cases | 0 | ,0 |
|  | Total | 1260 | 100,0 |
| Cases not selected | | 0 | ,0 |
| Total | | 1260 | 100,0 |

| a. If weighting is enabled, refer to the classification table for the total number of cases. |
| --- |

| **Coding of the dependent variable** | |
| --- | --- |
| Original value | Internal value |
| ,00 | 0 |
| 1,00 | 1 |

| **Coding of categorical variables** | | | |
| --- | --- | --- | --- |
|  | | Frequency | Parameter coding |
|  |  |  | (1) |
| ALT_LEVEL_OF CONCIOUSNESS | NO | 1155 | ,000 |
|  | YES | 105 | 1,000 |

**Block 0: Initial block**

| **Classification table^b^** | | | | | |
| --- | --- | --- | --- | --- | --- |
|  | Observed | | Forecast | | |
|  |  |  | HAPU | | Correct Percentage |
|  |  |  | ,00 | 1,00 |  |
| Step 0 | HAPU | ,00 | 1148 | 0 | 100,0 |
|  |  | 1,00 | 112 | 0 | ,0 |
|  | Overall percentage | |  |  | 91,1 |

| a. A constant is included in the model. |
| --- |
| b. The cut-off value is ,500 |

| **Variables in the equation** | | | | | | | |
| --- | --- | --- | --- | --- | --- | --- | --- |
|  | | B | E.T. | Wald | g | Next | Exp(B) |
| Step 0 | Constant | -2,327 | ,099 | 552,695 | 1 | ,000 | ,098 |

| **Variables not in the equation** | | | | | |
| --- | --- | --- | --- | --- | --- |
|  | | | Score | g | Next |
| Step 0 | Variables | ALT_LEVEL_OF CONCIOUSNESS (1) | ,057 | 1 | ,811 |
|  | Global statistics | | ,057 | 1 | ,811 |

**Block 1: Method = Enter**

| **Omnibus tests on model coefficients** | | | | |
| --- | --- | --- | --- | --- |
|  | | Chi square | g | Next |
| Step 1 | Step | ,056 | 1 | ,813 |
|  | Block | ,056 | 1 | ,813 |
|  | Model | ,056 | 1 | ,813 |

| **Model Overview** | | | |
| --- | --- | --- | --- |
| Step | -2 plausibility log | R square of Cox and Snell | Nagelkerke Square R |
| 1 | 755,842a | ,000 | ,000 |

| a. The estimate has ended in iteration number 5 because the parameter estimates have changed by less than ,001. |
| --- |

| **Classification table** | | | | | |
| --- | --- | --- | --- | --- | --- |
|  | Observed | | Forecast | | |
|  |  |  | HAPU | | Correct Percentage |
|  |  |  | ,00 | 1,00 |  |
| Step 1 | HAPU | ,00 | 1148 | 0 | 100,0 |
|  |  | 1,00 | 112 | 0 | ,0 |
|  | Overall percentage | |  |  | 91,1 |

| a. The cut-off value is ,500 |
| --- |

| **Variables in the equation** | | | | | | |
| --- | --- | --- | --- | --- | --- | --- |
|  | | B | E.T. | Wald | g | Next |
|  |  |  |  |  |  |  |
| Step 1a | ALT_LEVEL_OF CONCIOUSNESS (1) | ,083 | ,348 | ,057 | 1 | ,811 |
|  | Constant | -2,334 | ,104 | 506,765 | 1 | ,000 |

| **Variables in the equation** | | | | |
| --- | --- | --- | --- | --- |
|  | | Exp(B) | C. I. 95% for EXP(B) | |
|  |  |  | Lower | Superior |
| Step 1a | ALT_LEVEL_OF CONCIOUSNESS (1) | 1,087 | ,549 | 2,150 |
|  | Constant | ,097 |  |  |

| a. Variable(s) entered in step 1: ALT_LEVEL_OF CONCIOUSNESS . |
| --- |

**Logistical Regression**

[Data Set1] C:Users\epefe\OneDrive\Escritorio\Extra_USOIsabel Díaz\articulo\BBDD 1260.sav

| **Summary of case processing** | | | |
| --- | --- | --- | --- |
| Unweighted cases | | N | Percentage |
| Selected Cases | Included in the analysis | 1260 | 100,0 |
|  | Lost Cases | 0 | ,0 |
|  | Total | 1260 | 100,0 |
| Cases not selected | | 0 | ,0 |
| Total | | 1260 | 100,0 |

| a. If weighting is enabled, refer to the classification table for the total number of cases. |
| --- |

| **Coding of the dependent variable** | |
| --- | --- |
| Original value | Internal value |
| ,00 | 0 |
| 1,00 | 1 |

| **Coding of categorical variables** | | | |
| --- | --- | --- | --- |
|  | | Frequency | Parameter coding |
|  |  |  | (1) |
| ALT_BODY TEMPERATURE | NO | 1003 | ,000 |
|  | YES | 257 | 1,000 |

**Block 0: Initial block**

| **Classification table^b^** | | | | | |
| --- | --- | --- | --- | --- | --- |
|  | Observed | | Forecast | | |
|  |  |  | HAPU | | Correct Percentage |
|  |  |  | ,00 | 1,00 |  |
| Step 0 | HAPU | ,00 | 1148 | 0 | 100,0 |
|  |  | 1,00 | 112 | 0 | ,0 |
|  | Overall percentage | |  |  | 91,1 |

| a. A constant is included in the model. |
| --- |
| 1. The cut-off value is ,500 |

| **Variables in the equation** | | | | | | | |
| --- | --- | --- | --- | --- | --- | --- | --- |
|  | | B | E.T. | Wald | g | Next | Exp(B) |
| Step 0 | Constant | -2,327 | ,099 | 552,695 | 1 | ,000 | ,098 |

| **Variables not in the equation** | | | | | |
| --- | --- | --- | --- | --- | --- |
|  | | | Score | g | Next |
| Step 0 | Variables | ALT_BODY TEMPERATURE(1) | 1,604 | 1 | ,205 |
|  | Global statistics | | 1,604 | 1 | ,205 |

**Block 1: Method = Enter**

| **Omnibus tests on model coefficients** | | | | |
| --- | --- | --- | --- | --- |
|  | | Chi square | g | Next |
| Step 1 | Step | 1,532 | 1 | ,216 |
|  | Block | 1,532 | 1 | ,216 |
|  | Model | 1,532 | 1 | ,216 |

| **Model Overview** | | | |
| --- | --- | --- | --- |
| Step | -2 plausibility log | R square of Cox and Snell | Nagelkerke Square R |
| 1 | 754,366a | ,001 | ,003 |

| a. The estimate has ended in iteration number 5 because the parameter estimates have changed by less than ,001. |
| --- |

| **Classification table** | | | | | |
| --- | --- | --- | --- | --- | --- |
|  | Observed | | Forecast | | |
|  |  |  | HAPU | | Correct Percentage |
|  |  |  | ,00 | 1,00 |  |
| Step 1 | HAPU | ,00 | 1148 | 0 | 100,0 |
|  |  | 1,00 | 112 | 0 | ,0 |
|  | Overall percentage | |  |  | 91,1 |

| a. The cut-off value is ,500 |
| --- |

| **Variables in the equation** | | | | | | |
| --- | --- | --- | --- | --- | --- | --- |
|  | | B | E.T. | Wald | g | Next |
|  |  |  |  |  |  |  |
| Step 1a | ALT_BODY TEMPERATURE (1) | ,291 | ,230 | 1,595 | 1 | ,207 |
|  | Constant | -2,392 | ,114 | 440,541 | 1 | ,000 |

| **Variables in the equation** | | | | |
| --- | --- | --- | --- | --- |
|  | | Exp(B) | C. I. 95% for EXP(B) | |
|  |  |  | Lower | Superior |
| Step 1a | ALT_BODY TEMPERATURE (1) | 1,338 | ,852 | 2,101 |
|  | Constant | ,091 |  |  |

| a. Variable(s) entered in step 1: ALT_BODY TEMPERATURE. |
| --- |

**Logistical Regression**

| **Summary of case processing** | | | |
| --- | --- | --- | --- |
| Unweighted cases | | N | Percentage |
| Selected Cases | Included in the analysis | 1260 | 100,0 |
|  | Lost Cases | 0 | ,0 |
|  | Total | 1260 | 100,0 |
| Cases not selected | | 0 | ,0 |
| Total | | 1260 | 100,0 |

| a. If weighting is enabled, refer to the classification table for the total number of cases. |
| --- |

| **Coding of the dependent variable** | |
| --- | --- |
| Original value | Internal value |
| ,00 | 0 |
| 1,00 | 1 |

| **Coding of categorical variables** | | | |
| --- | --- | --- | --- |
|  | | Frequency | Parameter coding |
|  |  |  | (1) |
| INCONTINENCE | NO | 790 | ,000 |
|  | YES | 470 | 1,000 |

**Block 0: Initial block**

| **Classification table^,b^** | | | | | |
| --- | --- | --- | --- | --- | --- |
|  | Observed | | Forecast | | |
|  |  |  | HAPU | | Correct Percentage |
|  |  |  | ,00 | 1,00 |  |
| Step 0 | HAPU | ,00 | 1148 | 0 | 100,0 |
|  |  | 1,00 | 112 | 0 | ,0 |
|  | Overall percentage | |  |  | 91,1 |

| a. A constant is included in the model. |
| --- |
| b. The cut-off value is ,500 |

| **Variables in the equation** | | | | | | | |
| --- | --- | --- | --- | --- | --- | --- | --- |
|  | | B | E.T. | Wald | g | Next | Exp(B) |
| Step 0 | Constant | -2,327 | ,099 | 552,695 | 1 | ,000 | ,098 |

| **Variables not in the equation** | | | | | |
| --- | --- | --- | --- | --- | --- |
|  | | | Score | g | Next |
| Step 0 | Variables | INCONTINENCE(1) | 5,812 | 1 | ,016 |
|  | Global statistics | | 5,812 | 1 | ,016 |

**Block 1: Method = Enter**

| **Omnibus tests on model coefficients** | | | | |
| --- | --- | --- | --- | --- |
|  | | Chi square | g | Next |
| Step 1 | Step | 6,074 | 1 | ,014 |
|  | Block | 6,074 | 1 | ,014 |
|  | Model | 6,074 | 1 | ,014 |

| **Model Overview** | | | |
| --- | --- | --- | --- |
| Step | -2 plausibility log | R square of Cox and Snell | Nagelkerke Square R |
| 1 | 749,824a | ,005 | ,011 |

| a. The estimate has ended in iteration number 5 because the parameter estimates have changed by less than ,001. |
| --- |

| **Classification table** | | | | | |
| --- | --- | --- | --- | --- | --- |
|  | Observed | | Forecast | | |
|  |  |  | HAPU | | Correct Percentage |
|  |  |  | ,00 | 1,00 |  |
| Step 1 | HAPU | ,00 | 1148 | 0 | 100,0 |
|  |  | 1,00 | 112 | 0 | ,0 |
|  | Overall percentage | |  |  | 91,1 |

| a. The cut-off value is ,500 |
| --- |

| **Variables in the equation** | | | | | | | |
| --- | --- | --- | --- | --- | --- | --- | --- |
|  | | B | E.T. | Wald | g | Next | Exp(B) |
|  |  |  |  |  |  |  |  |
| Step 1a | INCONTINENCE(1) | -,530 | ,222 | 5,705 | 1 | ,017 | ,589 |
|  | Constant | -2,156 | ,117 | 341,513 | 1 | ,000 | ,116 |

| **Variables in the equation** | | | |
| --- | --- | --- | --- |
|  | | C. I. 95% for EXP(B) | |
|  |  | Lower | Superior |
| Step 1a | INCONTINENCE(1) | ,381 | ,909 |
|  | Constant |  |  |

| a. Variable(s) entered in step 1: INCONTINENCE. |
| --- |

**Logistical Regression**

| **Summary of case processing** | | | |
| --- | --- | --- | --- |
| Unweighted cases | | N | Percentage |
| Selected Cases | Included in the analysis | 1260 | 100,0 |
|  | Lost Cases | 0 | ,0 |
|  | Total | 1260 | 100,0 |
| Cases not selected | | 0 | ,0 |
| Total | | 1260 | 100,0 |

| a. If weighting is enabled, refer to the classification table for the total number of cases. |
| --- |

| **Coding of the dependent variable** | |
| --- | --- |
| Original value | Internal value |
| ,00 | 0 |
| 1,00 | 1 |

| **Coding of categorical variables** | | | |
| --- | --- | --- | --- |
|  | | Frequency | Parameter coding |
|  |  |  | (1) |
| SECONDARY_EFFECTS OF_TREATMENT | NO | 706 | ,000 |
|  | YES | 554 | 1,000 |

**Block 0: Initial block**

| **Classification table^b^** | | | | | |
| --- | --- | --- | --- | --- | --- |
|  | Observed | | Forecast | | |
|  |  |  | HAPU | | Correct Percentage |
|  |  |  | ,00 | 1,00 |  |
| Step 0 | HAPU | ,00 | 1148 | 0 | 100,0 |
|  |  | 1,00 | 112 | 0 | ,0 |
|  | Overall percentage | |  |  | 91,1 |

| a. A constant is included in the model. |
| --- |
| b. The cut-off value is ,500 |

| **Variables in the equation** | | | | | | | |
| --- | --- | --- | --- | --- | --- | --- | --- |
|  | | B | E.T. | Wald | g | Next | Exp(B) |
| Step 0 | Constant | -2,327 | ,099 | 552,695 | 1 | ,000 | ,098 |

| **Variables not in the equation** | | | | | |
| --- | --- | --- | --- | --- | --- |
|  | | | Score | g | Next |
| Step 0 | Variables | SECONDARY_EFFECTS_OF_TTº (1) | 34,019 | 1 | ,000 |
|  | Global statistics | | 34,019 | 1 | ,000 |

**Block 1: Method = Enter**

| **Omnibus tests on model coefficients** | | | | |
| --- | --- | --- | --- | --- |
|  | | Chi square | g | Next |
| Step 1 | Step | 37,358 | 1 | ,000 |
|  | Block | 37,358 | 1 | ,000 |
|  | Model | 37,358 | 1 | ,000 |

| **Model Overview** | | | |
| --- | --- | --- | --- |
| Step | -2 plausibility log | R square of Cox and Snell | Nagelkerke Square R |
| 1 | 718,540a | ,029 | ,065 |

| a. The estimate has ended in iteration number 6 because the parameter estimates have changed by less than ,001. |
| --- |

| **Classification table** | | | | | |
| --- | --- | --- | --- | --- | --- |
|  | Observed | | Forecast | | |
|  |  |  | HAPU | | Correct Percentage |
|  |  |  | ,00 | 1,00 |  |
| Step 1 | HAPU | ,00 | 1148 | 0 | 100,0 |
|  |  | 1,00 | 112 | 0 | ,0 |
|  | Overall percentage | |  |  | 91,1 |

| a. The cut-off value is ,500 |
| --- |

| **Variables in the equation** | | | | | | |
| --- | --- | --- | --- | --- | --- | --- |
|  | | B | E.T. | Wald | g | Next |
|  |  |  |  |  |  |  |
| Step 1a | SECONDARY_EFFECTS OF_TREATMENT | -1,386 | ,254 | 29,862 | 1 | ,000 |
|  | Constant | -1,898 | ,112 | 288,296 | 1 | ,000 |

| **Variables in the equation** | | | | |
| --- | --- | --- | --- | --- |
|  | | Exp(B) | C. I. 95% for EXP(B) | |
|  |  |  | Lower | Superior |
| Step 1a | SECONDARY_EFFECTS OF_TREATMENT (1) | ,250 | ,152 | ,411 |
|  | Constant | ,150 |  |  |

| a. Variable(s) entered in step 1: SECONDARY_EFFECTS OF_TREATMENT. |
| --- |

**Logistical Regression**

| **Summary of case processing** | | | |
| --- | --- | --- | --- |
| Unweighted cases | | N | Percentage |
| Selected Cases | Included in the analysis | 1260 | 100,0 |
|  | Lost Cases | 0 | ,0 |
|  | Total | 1260 | 100,0 |
| Cases not selected | | 0 | ,0 |
| Total | | 1260 | 100,0 |

| a. If weighting is enabled, refer to the classification table for the total number of cases. |
| --- |

| **Coding of the dependent variable** | |
| --- | --- |
| Original value | Internal value |
| ,00 | 0 |
| 1,00 | 1 |

| **Coding of categorical variables** | | | |
| --- | --- | --- | --- |
|  | | Frequency | Parameter coding |
|  |  |  | (1) |
| ALT_OF_SKIN SENSIVITY | NO | 789 | ,000 |
|  | YES | 471 | 1,000 |

**Block 0: Initial block**

| **Classification table^,b^** | | | | | |
| --- | --- | --- | --- | --- | --- |
|  | Observed | | Forecast | | |
|  |  |  | HAPU | | Correct Percentage |
|  |  |  | ,00 | 1,00 |  |
| Step 0 | HAPU | ,00 | 1148 | 0 | 100,0 |
|  |  | 1,00 | 112 | 0 | ,0 |
|  | Overall percentage | |  |  | 91,1 |

| a. A constant is included in the model. |
| --- |
| b. The cut-off value is ,500 |

| **Variables in the equation** | | | | | | | |
| --- | --- | --- | --- | --- | --- | --- | --- |
|  | | B | E.T. | Wald | g | Next | Exp(B) |
| Step 0 | Constant | -2,327 | ,099 | 552,695 | 1 | ,000 | ,098 |

| **Variables not in the equation** | | | | | |
| --- | --- | --- | --- | --- | --- |
|  | | | Score | g | Next |
| Step 0 | Variables | ALT_OF_SKIN SENSIVITY (1) | 6,163 | 1 | ,013 |
|  | Global statistics | | 6,163 | 1 | ,013 |

**Block 1: Method = Enter**

| **Omnibus tests on model coefficients** | | | | |
| --- | --- | --- | --- | --- |
|  | | Chi square | g | Next |
| Step 1 | Step | 6,001 | 1 | ,014 |
|  | Block | 6,001 | 1 | ,014 |
|  | Model | 6,001 | 1 | ,014 |

| **Model Overview** | | | |
| --- | --- | --- | --- |
| Step | -2 plausibility log | R square of Cox and Snell | Nagelkerke Square R |
| 1 | 749,898a | ,005 | ,011 |

| a. The estimate has ended in iteration number 5 because the parameter estimates have changed by less than ,001. |
| --- |

| **Classification table** | | | | | |
| --- | --- | --- | --- | --- | --- |
|  | Observed | | Forecast | | |
|  |  |  | HAPU | | Correct Percentage |
|  |  |  | ,00 | 1,00 |  |
| Step 1 | HAPU | ,00 | 1148 | 0 | 100,0 |
|  |  | 1,00 | 112 | 0 | ,0 |
|  | Overall percentage | |  |  | 91,1 |

| a. The cut-off value is ,500 |
| --- |

| **Variables in the equation** | | | | | | |
| --- | --- | --- | --- | --- | --- | --- |
|  | | B | E.T. | Wald | g | Next |
|  |  |  |  |  |  |  |
| Step 1a | ALT_OF_SKIN SENSIVITY (1) | ,490 | ,199 | 6,071 | 1 | ,014 |
|  | Constant | -2,534 | ,136 | 345,042 | 1 | ,000 |

| **Variables in the equation** | | | | |
| --- | --- | --- | --- | --- |
|  | | Exp(B) | C. I. 95% for EXP(B) | |
|  |  |  | Lower | Superior |
| Step 1a | ALT_OF_SKIN SENSIVITY (1) | 1,632 | 1,105 | 2,410 |
|  | Constant | ,079 |  |  |

| a. Variable(s) entered in step 1: ALT_OF_SKIN SENSIVITY. |
| --- |

**Logistical Regression**

| **Summary of case processing** | | | |
| --- | --- | --- | --- |
| Unweighted cases | | N | Percentage |
| Selected Cases | Included in the analysis | 1260 | 100,0 |
|  | Lost Cases | 0 | ,0 |
|  | Total | 1260 | 100,0 |
| Cases not selected | | 0 | ,0 |
| Total | | 1260 | 100,0 |

| 1. If weighting is enabled, refer to the classification table for the total number of cases. | | |
| --- | --- | --- |
| **Coding of the dependent variable** | |  |
| Original value | Internal value |  |
| ,00 | 0 |  |
| 1,00 | 1 |  |

| **Coding of categorical variables** | | | |
| --- | --- | --- | --- |
|  | | Frequency | Parameter coding |
|  |  |  | (1) |
| DAYS OF HOSPITAL STAY_ > 7 DAYS | ,00 | 417 | ,000 |
|  | 1,00 | 843 | 1,000 |

**Block 0: Initial block**

| **Classification table^,b^** | | | | | |
| --- | --- | --- | --- | --- | --- |
|  | Observed | | Forecast | | |
|  |  |  | HAPU | | Correct Percentage |
|  |  |  | ,00 | 1,00 |  |
| Step 0 | HAPU | ,00 | 1148 | 0 | 100,0 |
|  |  | 1,00 | 112 | 0 | ,0 |
|  | Overall percentage | |  |  | 91,1 |

| a. A constant is included in the model. |
| --- |
| b. The cut-off value is ,500 |

| **Variables in the equation** | | | | | | | | | | | | |
| --- | --- | --- | --- | --- | --- | --- | --- | --- | --- | --- | --- | --- |
|  | | | B | E.T. | Wald | | g | Next | | | Exp(B) | |
| Step 0 | Constant | | -2,327 | ,099 | 552,695 | | 1 | ,000 | | | ,098 | |
| **Variables not in the equation** | | | | | | | | | | | |  |
|  | | | | | | Score | | | g | Next | |  |
| Step 0 | Variables | DAYS OF HOSPITAL STAY_  > 7 DAYS (1) | | | | 27,809 | | | 1 | ,000 | |  |
|  | Global statistics | | | | | 27,809 | | | 1 | ,000 | |  |

**Block 1: Method = Enter**

| **Omnibus tests on model coefficients** | | | | |
| --- | --- | --- | --- | --- |
|  | | Chi square | g | Next |
| Step 1 | Step | 33,093 | 1 | ,000 |
|  | Block | 33,093 | 1 | ,000 |
|  | Model | 33,093 | 1 | ,000 |

| **Model Overview** | | | |
| --- | --- | --- | --- |
| Step | -2 plausibility log | R square of Cox and Snell | Nagelkerke Square R |
| 1 | 722,806a | ,026 | ,057 |

| a. The estimate has ended in iteration number 6 because the parameter estimates have changed by less than ,001. |
| --- |

| **Classification table** | | | | | |
| --- | --- | --- | --- | --- | --- |
|  | Observed | | Forecast | | |
|  |  |  | HAPU | | Correct Percentage |
|  |  |  | ,00 | 1,00 |  |
| Step 1 | HAPU | ,00 | 1148 | 0 | 100,0 |
|  |  | 1,00 | 112 | 0 | ,0 |
|  | Overall percentage | |  |  | 91,1 |

| 1. The cut-off value is ,500 | | | | |  |  |  |
| --- | --- | --- | --- | --- | --- | --- | --- |
| **Variables in the equation** | | | | | | | |
|  | | B | E.T. | Wald | | g | Next |
|  |  |  |  |  |  |  |  |
| Step 1a | DAYS OF HOSPITAL STAY_ > 7 DAYS (1) | 1,513 | ,312 | 23,578 | | 1 | ,000 |
|  | Constant | -3,519 | ,293 | 144,322 | | 1 | ,000 |

| **Variables in the equation** | | | | |
| --- | --- | --- | --- | --- |
|  | | Exp(B) | C. I. 95% for EXP(B) | |
|  |  |  | Lower | Superior |
| Step 1a | DAYS OF HOSPITAL STAY_  > 7 DAYS (1) | 4,542 | 2,466 | 8,367 |
|  | Constant | ,030 |  |  |

| a. Variable(s) entered in step 1: DAYS OF HOSPITAL STAY_> 7 DAYS (1) |
| --- |

**Logistical Regression**

| **Summary of case processing** | | | |
| --- | --- | --- | --- |
| Unweighted cases | | N | Percentage |
| Selected Cases | Included in the analysis | 1260 | 100,0 |
|  | Lost Cases | 0 | ,0 |
|  | Total | 1260 | 100,0 |
| Cases not selected | | 0 | ,0 |
| Total | | 1260 | 100,0 |

| a. If weighting is enabled, refer to the classification table for the total number of cases. |
| --- |

| **Coding of the dependent variable** | | |  |  |  |
| --- | --- | --- | --- | --- | --- |
| Original value | Internal value | |  |  |  |
| ,00 | 0 | |  |  |  |
| 1,00 | 1 | |  |  |  |
| **Coding of categorical variables** | | | | | |
|  | | | | Frequency | Parameter coding |
|  |  |  |  |  | (1) |
| INCOME_DAYS_mas14 | | ,00 | | 915 | ,000 |
|  |  | 1,00 | | 345 | 1,000 |

**Block 0: Initial block**

| **Classification table^,b^** | | | | | |
| --- | --- | --- | --- | --- | --- |
|  | Observed | | Forecast | | |
|  |  |  | UPP_intra | | Correct Percentage |
|  |  |  | ,00 | 1,00 |  |
| Step 0 | UPP_intra | ,00 | 1148 | 0 | 100,0 |
|  |  | 1,00 | 112 | 0 | ,0 |
|  | Overall percentage | |  |  | 91,1 |

| a. A constant is included in the model. | | | | | | |  |  |
| --- | --- | --- | --- | --- | --- | --- | --- | --- |
| b. The cut-off value is ,500 | | | | | | |  |  |
| **Variables in the equation** | | | | | | | | |
|  | | B | E.T. | Wald | g | Next | | Exp(B) |
| Step 0 | Constant | -2,327 | ,099 | 552,695 | 1 | ,000 | | ,098 |

| **Variables not in the equation** | | | | | |
| --- | --- | --- | --- | --- | --- |
|  | | | Score | g | Next |
| Step 0 | Variables | DAYS OF HOSPITAL STAY_> 14 DAYS (1) | 48,387 | 1 | ,000 |
|  | Global statistics | | 48,387 | 1 | ,000 |

**Block 1: Method = Enter**

| **Omnibus tests on model coefficients** | | | | |
| --- | --- | --- | --- | --- |
|  | | Chi square | g | Next |
| Step 1 | Step | 43,033 | 1 | ,000 |
|  | Block | 43,033 | 1 | ,000 |
|  | Model | 43,033 | 1 | ,000 |

| **Model Overview** | | | |
| --- | --- | --- | --- |
| Step | -2 plausibility log | R square of Cox and Snell | Nagelkerke Square R |
| 1 | 712,865a | ,034 | ,074 |

| a. The estimate has ended in iteration number 5 because the parameter estimates have changed by less than ,001. | | | | | |  |
| --- | --- | --- | --- | --- | --- | --- |
| **Classification table** | | | | | | |
|  | Observed | | Forecast | | | |
|  |  |  | HAPU | | Correct Percentage | |
|  |  |  | ,00 | 1,00 |  |  |
| Step 1 | HAPU | ,00 | 1148 | 0 | 100,0 | |
|  |  | 1,00 | 112 | 0 | ,0 | |
|  | Overall percentage | |  |  | 91,1 | |

| a. The cut-off value is ,500 |
| --- |

| **Variables in the equation** | | | | | | |
| --- | --- | --- | --- | --- | --- | --- |
|  | | B | E.T. | Wald | g | Next |
|  |  |  |  |  |  |  |
| Step 1a | DAYS OF HOSPITAL STAY_> 14 DAYS (1) | 1,332 | ,202 | 43,492 | 1 | ,000 |
|  | Constant | -2,851 | ,145 | 384,123 | 1 | ,000 |

| **Variables in the equation** | | | | |
| --- | --- | --- | --- | --- |
|  | | Exp(B) | I.C. 95% for EXP(B) | |
|  |  |  | Lower | Superior |
| Step 1a | DAYS OF HOSPITAL STAY_> 14 DAYS (1) | 3,790 | 2,551 | 5,632 |
|  | Constant | ,058 |  |  |

| a. Variable(s) entered in step 1: DAYS OF HOSPITAL STAY_> 14 DAYS. |
| --- |

**Logistical Regression**

| **Summary of case processing** | | | |
| --- | --- | --- | --- |
| Unweighted cases | | N | Percentage |
| Selected Cases | Included in the analysis | 1260 | 100,0 |
|  | Lost Cases | 0 | ,0 |
|  | Total | 1260 | 100,0 |
| Cases not selected | | 0 | ,0 |
| Total | | 1260 | 100,0 |

| a. If weighting is enabled, refer to the classification table for the total number of cases. |
| --- |

| **Coding of the dependent variable** | |
| --- | --- |
| Original value | Internal value |
| ,00 | 0 |
| 1,00 | 1 |

| **Coding of categorical variables** | | | |
| --- | --- | --- | --- |
|  | | Frequency | Parameter coding |
|  |  |  | (1) |
| DAYS OF HOSPITAL STAY_> 21 DAYS | ,00 | 1123 | ,000 |
|  | 1,00 | 137 | 1,000 |

**Block 0: Initial block**

| **Classification table^,b^** | | | | | |
| --- | --- | --- | --- | --- | --- |
|  | Observed | | Forecast | | |
|  |  |  | HAPU | | Correct Percentage |
|  |  |  | ,00 | 1,00 |  |
| Step 0 | HAPU | ,00 | 1148 | 0 | 100,0 |
|  |  | 1,00 | 112 | 0 | ,0 |
|  | Overall percentage | |  |  | 91,1 |

| a. A constant is included in the model. |
| --- |
| 1. The cut-off value is ,500 |

| **Variables in the equation** | | | | | | | |
| --- | --- | --- | --- | --- | --- | --- | --- |
|  | | B | E.T. | Wald | g | Next | Exp(B) |
| Step 0 | Constant | -2,327 | ,099 | 552,695 | 1 | ,000 | ,098 |

| **Variables not in the equation** | | | | | |
| --- | --- | --- | --- | --- | --- |
|  | | | Score | g | Next |
| Step 0 | Variables | DAYS OF HOSPITAL STAY_> 21 DAYS (1) | 72,751 | 1 | ,000 |
|  | Global statistics | | 72,751 | 1 | ,000 |

**Block 1: Method = Enter**

| **Omnibus tests on model coefficients** | | | | |
| --- | --- | --- | --- | --- |
|  | | Chi square | g | Next |
| Step 1 | Step | 52,025 | 1 | ,000 |
|  | Block | 52,025 | 1 | ,000 |
|  | Model | 52,025 | 1 | ,000 |

| **Model Overview** | | | |
| --- | --- | --- | --- |
| Step | -2 plausibility log | R square of Cox and Snell | Nagelkerke Square R |
| 1 | 703,873a | ,040 | ,090 |

| a. The estimate has ended in iteration number 5 because the parameter estimates have changed by less than ,001. |
| --- |

| **Classification table** | | | | | |
| --- | --- | --- | --- | --- | --- |
|  | Observed | | Forecast | | |
|  |  |  | HAPU | | Correct Percentage |
|  |  |  | ,00 | 1,00 |  |
| Step 1 | HAPU | ,00 | 1148 | 0 | 100,0 |
|  |  | 1,00 | 112 | 0 | ,0 |
|  | Overall percentage | |  |  | 91,1 |

| a. The cut-off value is ,500 |
| --- |

| **Variables in the equation** | | | | | | |
| --- | --- | --- | --- | --- | --- | --- |
|  | | B | E.T. | Wald | g | Next |
|  |  |  |  |  |  |  |
| Step 1a | DAYS OF HOSPITAL STAY_> 21 DAYS (1) | 1,745 | ,225 | 60,280 | 1 | ,000 |
|  | Constant | -2,666 | ,121 | 485,155 | 1 | ,000 |

| **Variables in the equation** | | | | |
| --- | --- | --- | --- | --- |
|  | | Exp(B) | C. I. 95% for EXP(B) | |
|  |  |  | Lower | Superior |
| Step 1a | DAYS OF HOSPITAL STAY_> 21 DAYS (1) | 5,724 | 3,685 | 8,892 |
|  | Constant | ,070 |  |  |

| a. Variable(s) entered in step 1: DAYS OF HOSPITAL STAY_> 21 DAYS. |
| --- |

**Logistical Regression**

| **Summary of case processing** | | | |
| --- | --- | --- | --- |
| Unweighted cases | | N | Percentage |
| Selected Cases | Included in the analysis | 1260 | 100,0 |
|  | Lost Cases | 0 | ,0 |
|  | Total | 1260 | 100,0 |
| Cases not selected | | 0 | ,0 |
| Total | | 1260 | 100,0 |

| a. If weighting is enabled, refer to the classification table for the total number of cases. |
| --- |

| **Coding of the dependent variable** | |
| --- | --- |
| Original value | Internal value |
| ,00 | 0 |
| 1,00 | 1 |

| **Coding of categorical variables** | | | |
| --- | --- | --- | --- |
|  | | Frequency | Parameter coding |
|  |  |  | (1) |
| Medical Wards | ,00 | 690 | ,000 |
|  | 1,00 | 570 | 1,000 |

**Block 0: Initial block**

| **Classification table^,b^** | | | | | |
| --- | --- | --- | --- | --- | --- |
|  | Observed | | Forecast | | |
|  |  |  | HAPU | | Correct Percentage |
|  |  |  | ,00 | 1,00 |  |
| Step 0 | HAPU | ,00 | 1148 | 0 | 100,0 |
|  |  | 1,00 | 112 | 0 | ,0 |
|  | Overall percentage | |  |  | 91,1 |

| a. A constant is included in the model. |
| --- |
| b. The cut-off value is ,500 |

| **Variables in the equation** | | | | | | | |
| --- | --- | --- | --- | --- | --- | --- | --- |
|  | | B | E.T. | Wald | g | Next | Exp(B) |
| Step 0 | Constant | -2,327 | ,099 | 552,695 | 1 | ,000 | ,098 |

| **Variables not in the equation** | | | | | |
| --- | --- | --- | --- | --- | --- |
|  | | | Score | g | Next |
| Step 0 | Variables | Medical_Wards (1) | 2,127 | 1 | ,145 |
|  | Global statistics | | 2,127 | 1 | ,145 |

**Block 1: Method = Enter**

| **Omnibus tests on model coefficients** | | | | |
| --- | --- | --- | --- | --- |
|  | | Chi square | g | Next |
| Step 1 | Step | 2,117 | 1 | ,146 |
|  | Block | 2,117 | 1 | ,146 |
|  | Model | 2,117 | 1 | ,146 |

| **Model Overview** | | | |
| --- | --- | --- | --- |
| Step | -2 plausibility log | R square of Cox and Snell | Nagelkerke Square R |
| 1 | 753,781a | ,002 | ,004 |

| a. The estimate has ended in iteration number 5 because the parameter estimates have changed by less than ,001. |
| --- |

| **Classification table** | | | | | |
| --- | --- | --- | --- | --- | --- |
|  | Observed | | Forecast | | |
|  |  |  | HAPU | | Correct Percentage |
|  |  |  | ,00 | 1,00 |  |
| Step 1 | HAPU | ,00 | 1148 | 0 | 100,0 |
|  |  | 1,00 | 112 | 0 | ,0 |
|  | Overall percentage | |  |  | 91,1 |

| a. The cut-off value is ,500 |
| --- |

| **Variables in the equation** | | | | | | | |
| --- | --- | --- | --- | --- | --- | --- | --- |
|  | | B | E.T. | Wald | g | Next | Exp(B) |
|  |  |  |  |  |  |  |  |
| Step 1a | Medical_Wards (1) | ,288 | ,198 | 2,116 | 1 | ,146 | 1,334 |
|  | Constant | -2,466 | ,142 | 302,736 | 1 | ,000 | ,085 |

| **Variables in the equation** | | | |
| --- | --- | --- | --- |
|  | | C. I. 95% for EXP(B) | |
|  |  | Lower | Superior |
| Step 1a | Medica_Wards(1) | ,905 | 1,968 |
|  | Constant |  |  |

| a. Variable(s) entered in step 1: Medical_Wards. |
| --- |

**Logistical Regression**

| **Summary of case processing** | | | |
| --- | --- | --- | --- |
| Unweighted cases | | N | Percentage |
| Selected Cases | Included in the analysis | 1260 | 100,0 |
|  | Lost Cases | 0 | ,0 |
|  | Total | 1260 | 100,0 |
| Cases not selected | | 0 | ,0 |
| Total | | 1260 | 100,0 |

| a. If weighting is enabled, refer to the classification table for the total number of cases. |
| --- |

| **Coding of the dependent variable** | |
| --- | --- |
| Original value | Internal value |
| ,00 | 0 |
| 1,00 | 1 |

| **Coding of categorical variables** | | | |
| --- | --- | --- | --- |
|  | | Frequency | Parameter coding |
|  |  |  | (1) |
| Surgical_Wards | ,00 | 1021 | ,000 |
|  | 1,00 | 239 | 1,000 |

**Block 0: Initial block**

| **Classification table^,b^** | | | | | |
| --- | --- | --- | --- | --- | --- |
|  | Observed | | Forecast | | |
|  |  |  | HAPU | | Correct Percentage |
|  |  |  | ,00 | 1,00 |  |
| Step 0 | HAPU | ,00 | 1148 | 0 | 100,0 |
|  |  | 1,00 | 112 | 0 | ,0 |
|  | Overall percentage | |  |  | 91,1 |

| a. A constant is included in the model. | | | | | | |  |  |
| --- | --- | --- | --- | --- | --- | --- | --- | --- |
| b. The cut-off value is ,500 | | | | | | |  |  |
| **Variables in the equation** | | | | | | | | |
|  | | B | E.T. | Wald | g | Next | | Exp(B) |
| Step 0 | Constant | -2,327 | ,099 | 552,695 | 1 | ,000 | | ,098 |

| **Variables not in the equation** | | | | | |
| --- | --- | --- | --- | --- | --- |
|  | | | Score | g | Next |
| Step 0 | Variables | Surgical_Wards (1) | ,196 | 1 | ,658 |
|  | Global statistics | | ,196 | 1 | ,658 |

**Block 1: Method = Enter**

| **Omnibus tests on model coefficients** | | | | |
| --- | --- | --- | --- | --- |
|  | | Chi square | g | Next |
| Step 1 | Step | ,193 | 1 | ,661 |
|  | Block | ,193 | 1 | ,661 |
|  | Model | ,193 | 1 | ,661 |

| **Model Overview** | | | |
| --- | --- | --- | --- |
| Step | -2 plausibility log | R square of Cox and Snell | Nagelkerke Square R |
| 1 | 755,705a | ,000 | ,000 |

| a. The estimate has ended in iteration number 5 because the parameter estimates have changed by less than ,001. | | | | | |  |
| --- | --- | --- | --- | --- | --- | --- |
| **Classification table** | | | | | | |
|  | Observed | | Forecast | | | |
|  |  |  | HAPU | | Correct Percentage | |
|  |  |  | ,00 | 1,00 |  |  |
| Step 1 | HAPU | ,00 | 1148 | 0 | 100,0 | |
|  |  | 1,00 | 112 | 0 | ,0 | |
|  | Overall percentage | |  |  | 91,1 | |

| a. The cut-off value is ,500 |
| --- |

| **Variables in the equation** | | | | | | | |
| --- | --- | --- | --- | --- | --- | --- | --- |
|  | | B | E.T. | Wald | g | Next | Exp(B) |
|  |  |  |  |  |  |  |  |
| Step 1a | Surgical_Wards (1) | ,109 | ,246 | ,196 | 1 | ,658 | 1,115 |
|  | Constant | -2,349 | ,111 | 448,161 | 1 | ,000 | ,095 |

| **Variables in the equation** | | | |
| --- | --- | --- | --- |
|  | | C.I. 95% for EXP(B) | |
|  |  | Lower | Superior |
| Step 1a | Surgical_Wards(1) | ,689 | 1,805 |
|  | Constant |  |  |

| a. Variable(s) entered in step 1: Surgical_Wards. |
| --- |

**Logistical Regression**

| **Summary of case processing** | | | |
| --- | --- | --- | --- |
| Unweighted cases | | N | Percentage |
| Selected Cases | Included in the analysis | 1260 | 100,0 |
|  | Lost Cases | 0 | ,0 |
|  | Total | 1260 | 100,0 |
| Cases not selected | | 0 | ,0 |
| Total | | 1260 | 100,0 |

| a. If weighting is enabled, refer to the classification table for the total number of cases. |
| --- |

| **Coding of the dependent variable** | |
| --- | --- |
| Original value | Internal value |
| ,00 | 0 |
| 1,00 | 1 |

| **Coding of categorical variables** | | | |
| --- | --- | --- | --- |
|  | | Frequency | Parameter coding |
|  |  |  | (1) |
| Medical_Surgical_Wards | ,00 | 809 | ,000 |
|  | 1,00 | 451 | 1,000 |

**Block 0: Initial block**

| **Classification table^b^** | | | | | |
| --- | --- | --- | --- | --- | --- |
|  | Observed | | Forecast | | |
|  |  |  | HAPU | | Correct Percentage |
|  |  |  | ,00 | 1,00 |  |
| Step 0 | HAPU | ,00 | 1148 | 0 | 100,0 |
|  |  | 1,00 | 112 | 0 | ,0 |
|  | Overall percentage | |  |  | 91,1 |

| a. A constant is included in the model. | | | | | | |  |  |
| --- | --- | --- | --- | --- | --- | --- | --- | --- |
| b. The cut-off value is ,500 | | | | | | |  |  |
| **Variables in the equation** | | | | | | | | |
|  | | B | E.T. | Wald | g | Next | | Exp(B) |
| Step 0 | Constant | -2,327 | ,099 | 552,695 | 1 | ,000 | | ,098 |

| **Variables not in the equation** | | | | | |
| --- | --- | --- | --- | --- | --- |
|  | | | Score | g | Next |
| Step 0 | Variables | Medical_Surgical_Wards (1) | 3,522 | 1 | ,061 |
|  | Global statistics | | 3,522 | 1 | ,061 |

**Block 1: Method = Enter**

| **Omnibus tests on model coefficients** | | | | |
| --- | --- | --- | --- | --- |
|  | | Chi square | g | Next |
| Step 1 | Step | 3,652 | 1 | ,056 |
|  | Block | 3,652 | 1 | ,056 |
|  | Model | 3,652 | 1 | ,056 |

| **Model Overview** | | | |
| --- | --- | --- | --- |
| Step | -2 plausibility log | R square of Cox and Snell | Nagelkerke Square R |
| 1 | 752,246a | ,003 | ,006 |

| a. The estimate has ended in iteration number 5 because the parameter estimates have changed by less than ,001. |
| --- |

| **Classification table** | | | | | |
| --- | --- | --- | --- | --- | --- |
|  | Observed | | Forecast | | |
|  |  |  | HAPU | | Correct Percentage |
|  |  |  | ,00 | 1,00 |  |
| Step 1 | HAPU | ,00 | 1148 | 0 | 100,0 |
|  |  | 1,00 | 112 | 0 | ,0 |
|  | Overall percentage | |  |  | 91,1 |

| a. The cut-off value is ,500 |
| --- |

| **Variables in the equation** | | | | | | |
| --- | --- | --- | --- | --- | --- | --- |
|  | | B | E.T. | Wald | g | Next |
|  |  |  |  |  |  |  |
| Step 1a | Medical_Surgical_Wards (1) | -,410 | ,220 | 3,483 | 1 | ,062 |
|  | Constant | -2,196 | ,117 | 351,458 | 1 | ,000 |

| **Variables in the equation** | | | | |
| --- | --- | --- | --- | --- |
|  | | Exp(B) | IIC. I. 95% for EXP(B) | |
|  |  |  | Lower | Superior |
| Step 1a | Medical_Surgical_Wards (1) | ,663 | ,431 | 1,021 |
|  | Constant | ,111 |  |  |

| a. Variable(s) entered in step 1: Medical_Surgical_Wards. |
| --- |
