# Supplementary material for: Incidence of hospital-acquired pressure ulcers in patients with "minimal risk" according to the "Norton-MI" scale
Source: PLoS One. 2020 Jan 8;15(1):e0227052. doi: 10.1371/journal.pone.0227052 (PMC6948734; doi:10.1371/journal.pone.0227052)
Supplement: S1 Table — (DOCX) [file pone.0227052.s003.docx]

GET

FILE='C:Users\Im. Diaz\Desktop\ELIA 1 5 2019\BBDD 1260.sav'.

DATASET NAME Data_set1 WINDOW=FRONT. DATASET ACTIVATE Data_set1.

CROSSTABS

/TABLES=HIGHEST_INCOME_DAYS_7_RR BY PATIENTS_WITH_UPP_INTRA

/FORMAT=AVALUE TABLES

/STATISTICS=CHISQ CORR RISK

/CELLS=COUNT COLUMN TOTAL

/COUNT ROUND CELL.

**Cross tables**

**Notes**

| Output created |  | 11-MAY-2019 20:44:03 |
| --- | --- | --- |
| Comments |  |  |
| Entrance | Facts | C: Users. Diaz\Desktop\ELIA 1 5 2019\BBDD 1260.sav |
|  | Active dataset | Data_set1 |
|  | Filter | <none> |
|  | Weighting | <none> |
|  | Segment file | <none> |
|  | No. of rows in the job data file | 1260 |
| Management of lost value | Definition of absence | User-defined missing values are treated as lost. |
|  | Cases used | The statistics for each table are based on all cases with valid data in the ranges specified for all variables in each table. |
| Syntax |  | CROSSTABS  /TABLES=SUPERIOR_INCOME_DAYS  &LT;FONT COLOR="#FFFF00"&GT;-==- SYNC:ßÇÈÂÈÂ  /FORMAT=AVALUE TABLES  /STATISTICS=CHISQ CORR RISK  /CELLS=COUNT COLUMN TOTAL  /COUNT ROUND CELL. |
| Resources | Processor time | 00:00:00,02 |
|  | Elapsed time | 00:00:00,02 |
|  | Dimensions requested | 2 |
|  | Available boxes | 131029 |

[Data_Set1] C:Users\Im. Diaz\Desktop\ELIA 1 5 2019\BBDD 1260.sav

**Case Processing Summary**

|  | Cases | | | | | |
| --- | --- | --- | --- | --- | --- | --- |
|  | Valid | | Lost | | Total | |
|  | N | Percentage | N | Percentage | N | Percentage |
| DAYS_OF_HOSPITAL_  STAY_SUPERIOR_7_RR * PATIENTS IN INTRA UPP PROTOCOL | 1260 | 100,0% | 0 | 0,0% | 1260 | 100,0% |

**DAYS_OF_HOSPITAL_ STAY_SUPERIOR_7_RRR*PACENT IN PROTOCOL HAPU cross tabulation**

|  | | | PATIENTS | | Total |
| --- | --- | --- | --- | --- | --- |
|  |  |  | WITH HAPU | WITHOUT HAPU |  |
| DAYS_OF_HOSPITAL_  STAY_SUPERIOR_7_RR | UPPER 7 DAYS | Counting | 100 | 743 | 843 |
|  |  | within PATIENTS IN PROTOCOL UPP INTRA | 89,3% | 64,7% | 66,9% |
|  |  | of total | 7,9% | 59,0% | 66,9% |
|  | REST | Counting | 12 | 405 | 417 |
|  |  | within PATIENTS IN PROTOCOL UPP INTRA | 10,7% | 35,3% | 33,1% |
|  |  | of total | 1,0% | 32,1% | 33,1% |
| Total |  | Counting | 112 | 1148 | 1260 |
|  |  | within PATIENTS IN PROTOCOL UPP INTRA | 100,0% | 100,0% | 100,0% |
|  |  | of total | 8,9% | 91,1% | 100,0% |

**Chi-square tests**

|  | Value | g | Sig. asymptotic (2 sides) | Exact meaning (2 sides) | Exact meaning (1 side) |
| --- | --- | --- | --- | --- | --- |
| Chi-square of Pearson | 27,809 | 1 | ,000 | ,000 | ,000 |
| Continuity correction | 26,710 | 1 | ,000 |  |  |
| Reason for plausibility | 33,093 | 1 | ,000 |  |  |
| Fisher's exact test |  |  |  |  |  |
| Linear association by linear | 27,787 | 1 | ,000 |  |  |
| N of valid cases | 1260 |  |  |  |  |

**Symmetrical measurements**

|  | | Value | Asymptotic standard error | Approx. S | Approx. Sig. |
| --- | --- | --- | --- | --- | --- |
| Interval per interval | R for person | ,149 | ,020 | 5,328 | ,000 |
| Ordinal by ordinal | Spearman Correlation | ,149 | ,020 | 5,328 | ,000 |
| N of valid cases |  | 1260 |  |  |  |

**Risk estimation**

|  | Value | 95% confidence interval | |
| --- | --- | --- | --- |
|  |  | Lower | Superior |
| Odds ratio for  DAYS_OF_HOSPITAL_ STAY_SUPERIOR_7_RR (SUPERIOR 7 DAYS / REST) | 4,542 | 2,466 | 8,367 |
| For cohort PATIENTS IN PROTOCOL UPP INTRA = WITH UPPI | 4,122 | 2,292 | 7,415 |
| For cohort PATIENTS IN PROTOCOL UPP INTRA = NO UPPI | ,907 | ,881 | ,935 |
| N of valid cases | 1260 |  |  |
